# Physiologically based pharmacokinetic (PBPK) model to predict the magnitude of drug-drug interaction between vincristine and the azole antifungals isavuconazole, posaconazole, and voriconazole in children

**DOI:** 10.1128/aac.00444-26

**Published:** 2026-07-22

**Authors:** Mary P. Choules, Amit Desai, Laura Lynn Kovanda, Yukio Otsuka, Roger J. Brüggemann, Peter L. Bonate

**Affiliations:** 1 Astellas Pharma Global, Inc. 508332 , Northbrook, Illinois, USA; 2 Astellas Pharma, Inc. 10873 , Tokyo, Japan; 3 Department of Pharmacy, Pharmacology and Toxicology, Radboud University Medical Center, RadboudUMC-CWZ Center of Expertise in Mycology 6034, Nijmegen, the Netherlands; Providence Portland Medical Center, Portland, Oregon, USA

**Keywords:** physiologically based pharmacokinetics (PBPK), drug-drug interaction (DDI), Simcyp, isavuconazole, posaconazole, voriconazole, vincristine, antifungal, pediatric

## Abstract

Invasive fungal diseases (IFDs), including invasive aspergillosis (IA) and invasive mucormycosis (IM), occur primarily in immunocompromised and/or hospitalized patients and are associated with increased morbidity and mortality. Isavuconazole and posaconazole are triazole compounds approved for the treatment of IA/IM in adults and children, while voriconazole is approved for IA treatment only. All triazole compounds inhibit CYP3A, and both isavuconazole and posaconazole inhibit the transporter P-gp. Pediatric patients being treated for leukemia with vincristine frequently experience fungal infections, requiring treatment. Vincristine is metabolized by CYP3A4 and CYP3A5 and eliminated via P-gp transport. The risk of drug-drug interactions (DDIs) between vincristine and triazole compounds is known; however, the magnitude of risk has not been quantified. In the present analysis, physiologically based pharmacokinetic (PBPK) models were built for the triazoles and vincristine in a pediatric cancer population using the Simcyp simulator and were used to predict the change in vincristine exposure following coadministration with triazoles. Different age groups and CYP3A5 phenotypes were also explored. Modeling predicted that vincristine geometric mean AUC_inf_ ratios in CYP3A5 poor metabolizers (PMs) ranged from 2.01- to 2.25-fold, 4.97- to 5.20-fold, and 1.43- to 1.53-fold with isavuconazole, posaconazole, and voriconazole, respectively. In CYP3A5 extensive metabolizers (EMs), vincristine AUC_inf_ ratios ranged from 1.22- to 1.38-fold, 1.19- to 1.24-fold, and 1.05- to 1.07-fold for isavuconazole, posaconazole, and voriconazole, respectively. The model predicted vincristine AUC_inf_ >100 ng·h/mL in CYP3A5 PM and <100 ng·h/mL in CYP3A5 EM; therefore, vincristine dose reduction may be required only in CYP3A5 null expressors.

## INTRODUCTION

Invasive fungal diseases (IFDs), such as invasive aspergillosis (IA) and invasive mucormycosis (IM), occur primarily in immunocompromised and/or hospitalized patients and are associated with morbidity and mortality, especially in pediatric populations (1). Unfortunately, pediatric patients being treated for leukemia with vincristine frequently experience invasive fungal infections, requiring treatment or prophylaxis with an antifungal agent. There are limited options available for the treatment of IA and IM. The top recommended treatment options include the following: voriconazole, isavuconazole, posaconazole, and liposomal amphotericin B (2). Three of the treatment options are triazole antifungal therapies (voriconazole, isavuconazole, and posaconazole), all of which suffer from a wide range of CYP and transporter-related drug-drug interactions (DDIs) (3, 4). The fourth treatment option, liposomal amphotericin B, is a formulation of amphotericin B with an improved safety profile compared to the original formulation; however, there are still nephrotoxicity risks and infusion-related reactions, and it is only available for iv administration (5).

Therapeutic drug monitoring (TDM) for vincristine is challenging; however, there are general recommendations that an area under the curve (AUC) of 50 to 100 ng·h/mL is considered safe and efficacious, with higher exposure increasing the risk of neurotoxicity and constipation (6–8). However, reliance on TDM alone is generally not recommended (9). One of the compounding factors is the sensitivity of vincristine exposure to the CYP3A5 phenotype. Studies have found that vincristine clearance can be up to 5-fold greater in CYP3A5 extensive metabolizers (EMs) than poor metabolizers (PMs) (10). Vincristine is also a P-gp substrate, with the prescribing information advising against co-administration with P-gp inhibitors.

Both posaconazole and voriconazole are classified as strong CYP3A4 inhibitors, while isavuconazole is classified as a moderate CYP3A4/5 inhibitor (11). In addition, posaconazole and isavuconazole are both P-gp inhibitors *in vitro* and *in vivo*, while voriconazole was not found to inhibit P-gp (12, 13). Considering that triazole antifungals inhibit the metabolism and/or elimination of vincristine (CYP3A and P-gp), it is important to consider the risks of their co-administration with vincristine. The increased risk of adverse events associated with triazole co-administration with vincristine is known and has been documented (8, 14).

However, there is still a significant gap in the true understanding of the magnitude of DDIs between vincristine and triazole antifungals. In addition, this gap in understanding extends to CYP3A pharmacogenomics and the impact it may have on the resulting DDI (15, 16). Considering that antifungal use with vincristine administration can be difficult to avoid in situations of IA and IM, understanding the magnitude of increase in vincristine exposure with these compounds and how it relates to vincristine toxicity is clinically imperative. Due to the toxicity of vincrisitine, clinical controlled DDI studies are ethically difficult to conduct, especially in pediatric populations. Physiologically based pharmacokinetic (PBPK) modeling has been historically implemented to predict the magnitude of DDI for medication combinations where clinical DDI studies may be difficult to conduct (17, 18). In the present analysis, we report on the predicted magnitude of DDI when triazole compounds (isavuconazole, posaconazole, and voriconazole) are co-administered with vincristine in a pediatric cancer population and extrapolate those results to their clinical implications.

## RESULTS

**Fig 1:**
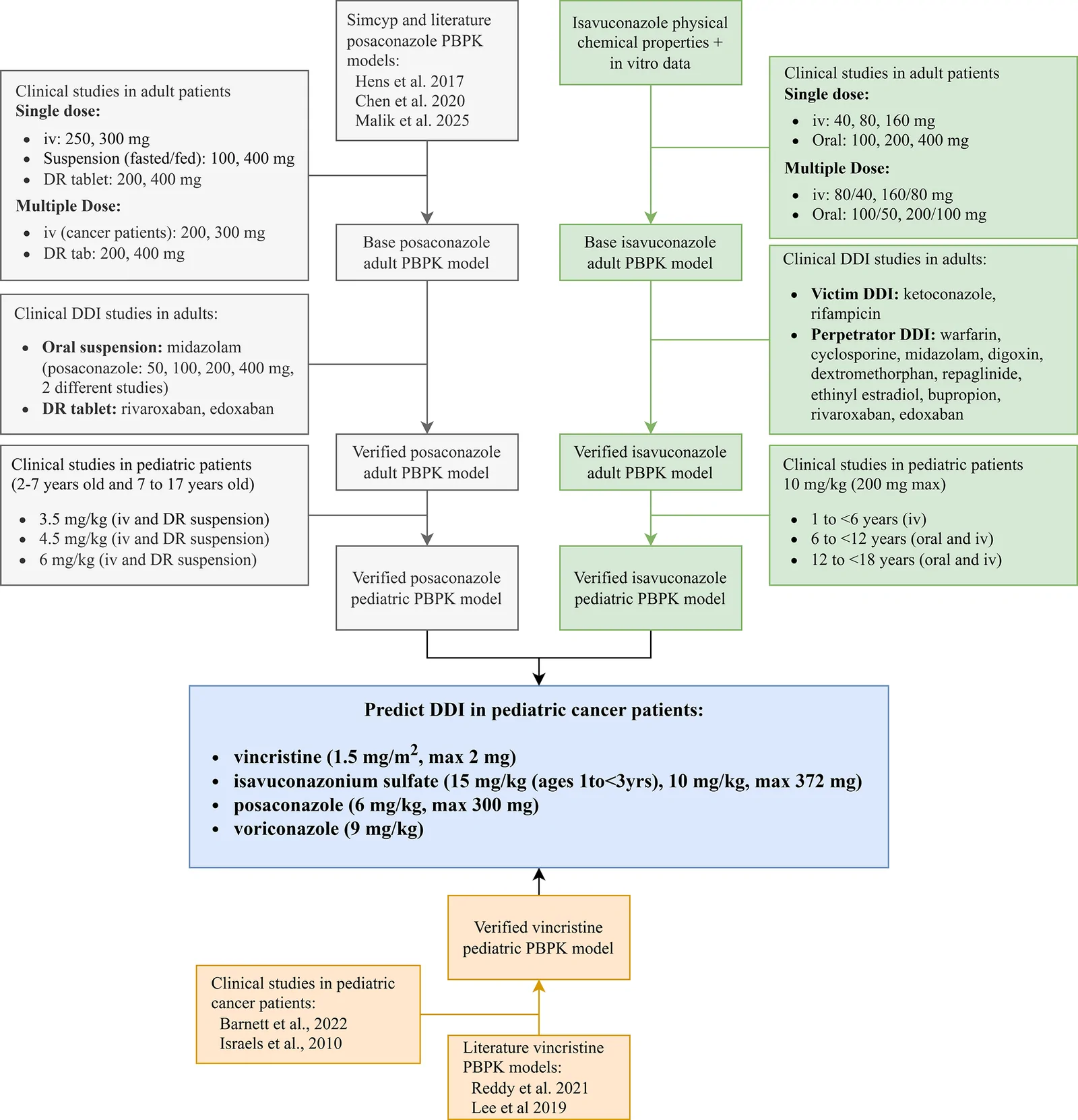
Overall workflow for the building, verification, and implementation of vincristine DDI interaction with triazoles.

The performance of the isavuconazole PBPK was described previously (manuscript submitted for peer review). Information about the other PBPK models utilized herein can be found in the supplemental information. An overview of the workflow for the building, verification, and implementation of the PBPK modeling can be found in Fig. 1.

### Prediction of DDI between triazoles and vincristine in pediatric cancer patients

The AUC_inf_ ratios with the 90% CI of vincristine in the presence of isavuconazole, posaconazole, or voriconazole predicted by age group or CYP3A5 metabolism status are listed in Table 1. In the EM population, the predicted vincristine AUC_inf_ ratio ranged from 1.22- to 1.38-fold, 1.19- to 1.24-fold, and 1.05- to 1.07-fold for isavuconazole, posaconazole, and voriconazole, respectively. In the PM population, the predicted vincristine AUC_inf_ ratio ranged from 1.94- to 2.25-fold, 4.97- to 5.20-fold, and 1.43- to 1.53-fold for isavuconazole, posaconazole, and voriconazole, respectively. The DDI risk from highest to lowest risk was determined to be as follows: posaconazole (PM), isavuconazole (PM), voriconazole (PM), isavuconazole (EM), posaconazole (EM), and voriconazole (EM).

**TABLE 1:** Model-predicted magnitude of DDIs between isavuconazole, posaconazole, or voriconazole with vincristine^*a*^

		1 to < 3 years		1 to < 6 years		6 to < 12 years		12 to < 18 years	
	CYP3A5 status	PM	EM	PM	EM	PM	EM	PM	EM
Isavuconazole	Inhibitor AUC_24_ (ng·h/mL)	121,000	117,000	82,800	79,900	92,500	89,300	79,400	76,000
	Inhibitor C_max_ (ng/mL)	10,600	10,400	7,140	7,000	7,500	7,340	6,290	6,130
	Inhibitor C_min_ (ng/mL)	3,750	3,580	2,580	2,470	2,970	2,840	2,680	2,550
	Vincristine AUC_inf_no inhibition (ng·h/mL)	86.7	44.3	85.1	43.8	83.0	43.1	70.8	36.4
	Vincristine AUC_inf_with inhibition (ng·h/mL)	195	61.1	171	56.3	168	54.7	138	44.5
	Vincristine AUC_inf_ ratio	2.25 (2.14, 2.36)	1.38 (1.35, 1.41)	2.01 (1.92, 2.10)	1.29 (1.26, 1.31)	2.02 (1.93, 2.12)	1.27 (1.25, 1.29)	1.94 (1.86, 2.04)	1.22 (1.20, 1.25)
Posaconazole	Inhibitor AUC_24_ (ng·h/mL)			30,700	31,400	32,000	32,800	35,600	36,400
	Inhibitor C_max_ (ng/mL)			3,270	3,290	3,330	3,340	3,320	3,370
	Inhibitor C_min_ (ng/mL)			780	813	806	843	1,000	1,030
	Vincristine AUC_inf_no inhibition (ng·h/mL)			86.5	44.3	84.3	43.5	69.5	36.5
	Vincristine AUC_inf_with inhibition (ng·h/mL)			443	55.0	410	52.7	345	43.8
	Vincristine AUC_inf_ ratio			5.20 (4.92, 5.30)	1.24 (1.22, 1.26)	4.68 (4.68, 5.05)	1.21 (1.19, 1.23)	4.97 (4.76, 5.18)	1.19 (1.17, 1.22)
Voriconazole	Inhibitor AUC_12_ (ng·h/mL)			54,100	55,300	69,500	70,800	83,500	82,400
	Inhibitor C_max_ (ng/mL)			6,860	6,970	8,780	8,900	10,800	10,700
	Inhibitor C_min_ (ng/mL)			2,850	2,920	3,940	4,010	4,700	4,600
	Vincristine AUC_inf_no inhibition (ng·h/mL)			86.8	44.2	85.0	43.5	70.8	36.4
	Vincristine AUC_inf_with inhibition (ng·h/mL)			133	47.4	127	46.2	101	38.3
	Vincristine AUC_inf_ ratio								
	Vincristine AUC_inf_ ratio			1.53 (1.47, 1.58)	1.07 (1.07, 1.08)	1.49 (1.44, 1.54)	1.06 (1.06, 1.07)	1.43 (1.38, 1.48)	1.05 (1.05, 1.06)

^*a*^ EM, extensive metabolizer; PM, poor metabolizer; AUC_24_, area under the curve from 0 to 24 h; AUC_inf_, area under the curve from 0 to infinity; C_max_, maximum concentration; C_min_, minimum or trough concentration; AUC ratio, AUC (with inhibitor) / AUC(without inhibitor). AUC ratio presented as geometric mean ratio (90% confidence interval).

**Fig 2:**
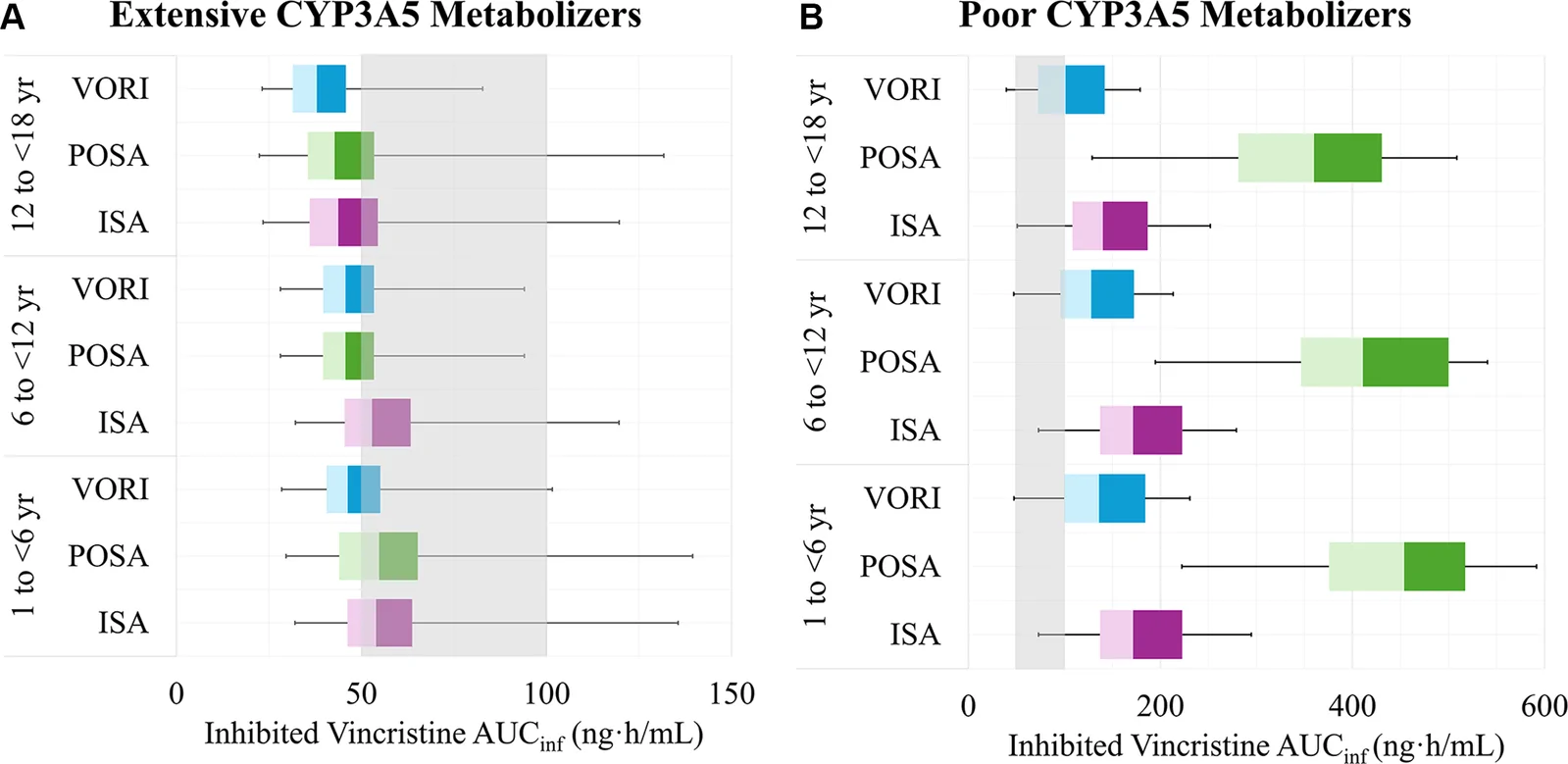
Simulated inhibited vincristine AUC_inf_ in CYP3A5 poor and extensive metabolizers stratified by the pediatric age group and triazole compound. VORI: voriconazole, POSA: posaconazole, ISA: isavuconazole, yr: year. The light gray box represents the estimated vincristine therapeutic window of 50 to ≤ 100 ng·h/mL. (A) extensive CYP3A5 metabolizers, (B) poor CYP3A5 metabolizers.

A box and whisker plot of the inhibited vincristine AUC_inf_ with an approximated vincristine therapeutic window indicated can be found in Fig. 2. In this plot, the simulations conducted in the EM population fell within the designated vincristine therapeutic window, except for voriconazole in the 12 to <18 year cohort. In contrast, all simulations conducted in the PM populations predicted vincristine exposure above the suggested upper limit of 100 ng·h/mL. There was no impact on the C_max_ ratio for any simulation due to the rapid bolus (1 to 5 min) administration of vincristine. Therefore, for simplicity, the parameter was not included in the table or figure. Triazole AUC_24/12h_, C_max_, and C_min_ at the time of vincristine dosing (day 7) were also provided. Based on the simulations conducted, vincristine uninhibited AUC_inf_ in the EM populations were ~2-fold lower than in the PM populations. In addition, isavuconazole exposure was slightly lower in the EM populations than in the PM populations. The CYP3A5 phenotype had no impact on posaconazole or voriconazole exposure.

### Sensitivity analysis

Specific results from the sensitivity analysis can be found in the supplemental information (Tables S19 and S20). In short, the DDI of vincristine was sensitive to the values of K_i,CYP3A4_ in isavuconazole and posaconazole PBPK models and to the values of V_max,CYP3A_ and CL_int,P-gp-liver_ in the vincristine model. The predicted vincristine AUC ratio with isavuconazole and posaconazole increased approximately 67% (3.25-fold) and 29% (6.68-fold), respectively, when the K_i,CYP3A4_ value was decreased to 10-fold lower than the default values used in the models. The DDI of vincristine was sensitive to the value of K_i,CYP3A5_ in the isavuconazole model, with the AUC ratio only increasing to approximately 69% (2.10-fold). The DDI of vincristine was also minimally sensitive to the values of K_i,P-gp_, and the AUC ratio for isavuconazole and posaconazole increased to approximately 18% (2.26-fold) and 5% (5.4-fold), respectively.

The vincristine DDI with posaconazole was sensitive to the value of CL_int,P-gp,liver_ in the vincristine model, with the AUC ratio increasing 70% to 8.89-fold when the value was increased 10-fold higher (45 µL/min/million cells) than the default value (4.5 µL/min/million cells). The vincristine DDI with posaconazole was sensitive to the value of V_max,CYP3A,_ with greater sensitivity to V_max,CYP3A5_ than to V_max,CYP3A4_. The AUC ratio decreased when the value of V_max,CYP3A4_ was increased or decreased, indicating shunting of vincristine clearance through CYP3A4 or through alternative pathways. The vincristine AUC ratio increased 146% when V_max,CYP3A5_ was decreased to 1.10 pmol/min/pmol isoform.

## DISCUSSION

### PBPK model evaluation

When assessing the PBPK model performances, geometric mean fold error (GMFE) for PK assessment and the Guest method (19) for DDI assessment were used. The model GMFE for all adult PBPK models was <1.5 (posaconazole: Table S5, vincristine: Table S11, edoxaban: Table S14) for all reported PK parameters. K_i_ and/or Ind_max_ values of CYP3A4, CYP3A5, and P-gp for all PBPK models were considered well verified. The multiple observed clinical DDI studies affecting these enzymes and/or transporters allowed for optimization and subsequent verification on different data sets. For the intended purpose, the K_i_ values for CYP3A4, CYP3A5, and P-gp are the essential parameters required for DDI prediction with vincristine and therefore were included in the sensitivity analysis.

When translating the model from adults to pediatric cancer populations, the overall model GMFE was <1.25 for all PK parameters and all PBPK compound models (isavuconazole and posaconazole: Table S7, voriconazole: Table S8, and vincristine: Table S11), except for voriconazole C_min,_ which was 1.27. The slightly higher GMFE for voriconazole C_min_ is influenced by the large variability in the observed C_min,_ which is clear from the reported range of values. Overall, PBPK model performance in the pediatric cancer populations was determined to be good and appropriate for predictions with vincristine.

### Model limitations

Initial model construction and verification were done in healthy adult subjects, while the DDI prediction was performed in pediatric cancer patients. Additional verification of CYP and transporter inhibition/induction parameters could not be performed in pediatric cancer patients due to the lack of available data. It is reasonable to assume that these values will not change significantly based on the population and are instead innate to the compound. This conclusion is further substantiated by the definition of a K_i_ value, which is the dissociation constant describing the binding affinity between the inhibitor and the enzyme. Therefore, unless the binding pocket of the given enzyme changes with the population, it is unlikely that the population would affect the model-determined K_i_ value. In addition, PBPK is uniquely suited to extrapolating DDI risk from adults to pediatric populations and has been implemented with great success in many situations, as detailed in the review by Johnson TN et al. (20). Therefore, although the inaccuracy of the predicted DDI can never be fully ruled out, there is good confidence in the predicted DDI.

Finally, the pediatric population model provided within the simulator uses adult anatomical and physiological data for the permeability-limited liver model. It is unknown if changes need to be made to adjust the permeability-limited liver model from adults to pediatric participants. This difference could affect P-gp transport of vincristine in the current model. However, considering the model captures the observed exposure of vincristine in pediatric cancer patients well, it was determined that the model was appropriate for the present application.

Overall, the sensitivity analysis indicated that changes in K_i_ and vincristine elimination parameters could alter model outcomes. In addition, the vincristine DDI with isavuconazole was more sensitive to changes in the model parameters than posaconazole. Sensitivity of the isavuconazole parameters might be attributed to the logistic growth in the magnitude of DDI as the maximum effect is observed (i.e., plateau effect). There was good confidence in the K_i,CYP3A4_ used in both isavuconazole and posaconazole models due to the considerable number of DDI studies with CYP3A4 involvement utilized during model development. For P-gp, there were fewer clinical studies available, which garnered less confidence. However, considering that both models were only marginally sensitive to a 10-fold decrease in K_i,P-gp_, the confidence in the relative DDI risk reported herein increases. Based on the sensitivity analysis, it appears that both isavuconazole and posaconazole are close to a clinically maximal inhibitory effect on P-gp. Unsurprisingly, the vincristine DDI results were sensitive to the values of V_max,CYP3A5_ and CL_int,P-gp,liver_. However, 10-fold increases or decreases in these parameters were easily ruled out due to supporting evidence from total body clearance in clinical studies and the mass balance study. Small changes in these parameters might be feasible; however, the sensitivity analysis indicated that small changes in these parameters would have a minimal impact on the predicted AUC ratio and would not affect the clinical conclusions drawn from the model predictions. Additionally, the parameters utilized in the vincristine model were verified using literature-reported data and mass balance data, lending to further confidence in the included parameters.

### Prediction of DDIs between triazoles and vincristine

The increased risk for adverse events when triazole compounds are co-administered with vincristine is well known and documented. In a retrospective analysis conducted by Huang D, et al., adverse events associated with the concomitant use of triazoles with vincristine were reported for itraconazole, posaconazole, and voriconazole: 66.7%, 28.6%, and 4.8%, respectively (14). Patients who received a two-drug combination experienced adverse events within 1 month of receiving treatment in 88.1% of cases (14). The study did not report on isavuconazole and mentioned that the frequency of events reported was too low due to its shorter time on the market but that isavuconazole, being a moderate CYP3A4 inhibitor, might offer a promising triazole combination with vincristine. In another study conducted by van Schie, et al., 20 pediatric patients were retrospectively assessed for vincristine toxicity with and without triazole co-medication (8). The study found significantly more constipation and neurotoxicity in patients receiving combination therapy. In addition, central nervous system (CNS) toxicity was only observed in those receiving combination therapy. To translate the clinical adverse event data to quantitative exposure data, the analysis discussed herein was conducted.

One must note the importance of the CYP3A phenotype on the overall exposure of CYP3A-metabolized drugs and the impact it may have on DDI risk. CYP3A represents the most abundantly expressed drug-metabolizing enzyme in the human liver and is responsible for metabolizing 30%–50% of clinical drugs (21). CYP3A4 and CYP3A5 are both located on chromosome 7 and share structural similarities; however, CYP3A5 has a higher and narrower active site, which contributes to its greater selectivity (21). Subjects with CYP3A5 genotype *3/*3 represent null expressors and are considered PMs through CYP3A5. This means that these subjects will have little to no CYP3A5 activity. More specifically, it is the CYP3A5*3 allele that leads to CYP3A5 inactivity due to the premature stop codon (21). Subjects with a genotype of *1/*1 allele combination are expressors or EMs through CYP3A5. However, there are other diplotype combinations that can lead to a range of CYP3A5 activities, such as *1/*3 (22). Due to the vast number of drugs metabolized by CYP3A4 and CYP3A5, guidance has started emerging suggesting running a minimum or extended variant allele panel to help guide drug dosing recommendations (22). Defining the CYP phenotype is important, especially for subjects who are CYP3A5 expressors as they will have lower total exposure of any drug with higher affinity for CYP3A5, like vincristine.

For all the simulations listed, the triazole trough concentrations fell within therapeutic goal concentrations for either treatment or prophylaxis. For reference, the goal trough concentrations in children are as follows: isavuconazole 2.5–5.0 mg/L (2,500–5,000 ng/mL) (23) for treatment, posaconazole >1 mg/L (1,000 ng/mL) for treatment or >0.7 mg/L (700 ng/mL) for prophylaxis (24), and voriconazole 1 to 5.5 mg/L (1,000–5,500 ng/mL) for treatment or prophylaxis (25). Isavuconazole can also be monitored by steady-state AUC_24_ with a goal AUC_24_ >60 mg·h/L (60,000 ng·h/mL) and with an upper exposure limit of 233 mg·h/L (233,000 ng·h/mL), which was also achieved for all simulations conducted. The posaconazole simulations used the recommended dose for the treatment of IA (6 mg/kg iv Q12H for the first day and then 6 mg/kg daily); however, C_min_ posaconazole concentrations were closer to goal prophylaxis levels instead of treatment levels. Therefore, the magnitude of DDIs from posaconazole inhibition will most likely be larger when trough levels are higher. Representative simulations were conducted with a posaconazole dose of 8 mg/kg in CYP3A5 expressors and null expressors aged 1 to <6 years old, which resulted in a C_min_ (trough) concentration of 1.1 mg/L (1,100 ng/mL) and the vincristine GM AUC_inf_ ratio of 5.67-fold (90% CI: 5.46–5.89) and 1.25-fold (90% CI: 1.23–1.27) in PM and EM, respectively. Increasing the posaconazole dose from 6 mg/kg to 8 mg/kg increased the inhibited vincristine AUC_inf_ by 11% and 0.5% in PM and EM, respectively. An increase in the magnitude of DDIs was expected, and based on this analysis, trough concentrations of posaconazole >1 mg/L are expected to increase vincristine GM AUC_inf_ by ~5- to 6-fold and ~1.19- to 1.25-fold in CYP3A5 PM and EM, respectively.

This trend of a higher magnitude of DDIs in the CYP3A5 null expressors vs the expressors was observed for all tested triazoles. Of the tested triazoles, voriconazole led to the smallest increase in vincristine AUC_inf_ since voriconazole does not inhibit P-gp, unlike isavuconazole and posaconazole. Posaconazole led to the largest increase in vincristine AUC_inf,_ and isavuconazole increased AUC_inf_ marginally more than voriconazole. This is consistent with the regulatory agency inhibitor classifications mentioned previously. In addition, the results also seem to agree with those of the meta-analysis presented previously, where reported vincristine AEs were lowest with voriconazole (14). When analyzing inhibited vincristine AUC_inf_, all triazoles in the PM populations resulted in a vincristine AUC_inf_ > 100 ng·h/mL when the dose of 1.5 mg/m^2^ was used across all simulations. Vincristine AUC >100 ng·h/mL has been linked to increased potential for toxicity; therefore, vincristine dose reduction may be needed in CYP3A5 null expressors, regardless of the triazole utilized. To explore the required dose reduction, a representative simulation was conducted with 50% vincristine dose reduction (0.75 mg/m^2^) with co-administration with isavuconazole in a PM population aged 1 to <6 years old. The model predicted an inhibited vincristine GM AUC_inf_ of 85.6 ng·h/mL, which falls below the 100 ng·h/mL toxicity threshold. For posaconazole, with a 5-fold vincristine dose reduction (0.3 mg/m^2^), the model predicted an inhibited vincristine GM AUC_inf_ of 82.3 ng·h/mL.

Interestingly, all three triazole antifungal agents were predicted to be viable options when the subject is a CYP3A5 EM. This may be due to a combination of vincristine’s preferential metabolism through CYP3A5 and the lower exposure of vincristine in this virtual population. To explore this hypothesis further, a representative simulation with isavuconazole in subjects 6 to <12 years old was conducted with 2× the vincristine dose. The recommended vincristine dose (1.5 mg/kg) and maximum dose (2 mg) were established from observed vincristine toxicity as higher doses and exposures. However, the uninhibited vincristine AUC_inf_ in the EM population was ~50% lower than that in the PM population and was below the recommended 50 ng·h/mL therapeutic threshold. Therefore, to mimic vincristine exposure captured in the PM population, a vincristine dose of 3 mg/m^2^ (with no dose maximum) was selected. Even though the uninhibited AUC_inf_ at 3 mg/m^2^ was the same as the PM population (at 1.5 mg/m^2^), the resulting AUC_inf_ ratio was identical to the original 1.5 mg/m^2^ (dose max 2 mg) in the EM population. These results indicate that the lower AUC ratio simulated in the EM populations is a result of CYP3A5’s greater capacity to metabolize vincristine over CYP3A4. This observation might also indicate that vincristine dosing may need to be increased in patients with CYP3A5 EM designation.

This trend may also be extrapolated to heterogeneous CYP3A5 expressors, for example, genotype *1/*3. Although heterogeneous populations were not explored in the present analysis, the homogeneous PM and EM phenotypes represent the worst-case and best-case DDI risk, respectively. Therefore, it is reasonable to assume that DDI risk for a heterogeneous CYP3A5 expressor would fall somewhere in the middle of the two representative cases explored herein. The impact of the CYP3A5 genotype and phenotype on DDI potential gives further support to the recommendations for patient CYP genotyping prior to treatment.

According to the sensitivity analysis, P-gp contribution and inhibition play a significant role in the vincristine AUC ratio. The impact of P-gp was highlighted by comparing the vincristine AUC ratio between voriconazole and posaconazole inhibition. Voriconazole and posaconazole are both strong CYP3A4 inhibitors; however, the magnitude of DDI is significantly higher with posaconazole due to P-gp inhibition. In addition, although isavuconazole is a moderate CYP3A inhibitor, P-gp inhibition increases the vincristine AUC ratio when compared to voriconazole. It is possible that the vincristine model overestimates the contribution of P-gp elimination, and therefore the model overestimated the potential DDI, but the sensitivity analysis explored this potential impact. One potential unintended benefit of triazole-based P-gp inhibition is reversing vincristine resistance through inhibition of tumor-specific P-gp transporters. However, that aspect would not be the focus of triazole therapy, and this application falls outside the scope of the present analysis but might be worth exploring further (26).

Other clinical factors to consider are the patient-dependent confounding factors such as inflammation, mucositis, altered hepatic function, and/or hypoalbuminemia. Pediatric cancer patients, especially those with active systemic fungal infections, commonly experience these comorbidities (27). Many of these conditions can alter the pharmacokinetic properties of a drug, from decreased absorption (28) to changes in CYP or transporter activity (29) or changes in drug distribution (30). Changes in clearance and volume of distribution between healthy volunteers and patients were accounted for in the posaconazole model (supplemental information). No changes for clearance or distribution appeared necessary for voriconazole or isavuconazole based on the data utilized; however, potential differences cannot be completely ruled out due to the nature of clinical trial inclusion and exclusion criteria. Use of the iv formulation in the DDI predictions removed any decrease in absorption due to intestinal mucositis. If an oral formulation of the triazole is used in these patients, decreased absorption may lead to decreased efficacy of the triazole, but it will decrease the DDI risk with vincristine. Changes in CYP activities due to inflammatory status were not explored in the present analysis; however, DDI risk may increase in patients with chronic inflammation due to the downregulation of CYP3A and P-gp activity (29, 31).

Based on the results of the predicted inhibited vincristine AUC_inf_, co-administration of vincristine with any triazole in a CYP3A5 null expressor would require vincristine dose reduction. The modeling suggests that the required vincristine dose adjustment is triazole-dependent. For posaconazole, based on the predicted >5-fold increase in AUC, best practice would be to avoid posaconazole co-administration with vincristine whenever possible. If avoidance is not possible, PBPK simulations suggest that a 5-fold reduction in vincristine dose (i.e., 0.3 mg/m^2^) may be required. For isavuconazole or voriconazole, coupled with TDM of triazole and vincristine trough concentrations, modeling predictions suggest that an initial conservative approach of 50% reduction in vincristine dose may be required (i.e., 0.75 mg/m^2^). However, based on some TDM guidelines, reliance on vincristine trough concentrations alone is not recommended, and the totality of patient presentation should be considered (9). If CYP3A phenotype data are unknown and TDM guidance is not possible, a conservative approach would be to assume the patient is a CYP3A5 null expressor and to start with a 50% reduction in vincristine dose with isavuconazole and voriconazole co-administration and a 5-fold reduction in vincristine dose for posaconazole co-administration. With this conservative approach, monitoring patient presentation for any emerging adverse events and clinical markers of efficacy becomes crucial.

The present analysis represents another application where PBPK was utilized to fill a gap in therapeutic knowledge where clinical trials may not be easily feasible. In addition, it provides further support and evidence for patient CYP genotyping and a call for increasing use of personalized medicine approaches to improve patient outcomes.

## MATERIALS AND METHODS

All simulations were carried out in the Simcyp Simulator (Certara, Sheffield, UK, version 24). Literature-referenced plasma concentration data were extracted using GetData Graph Digitizer (http://getdata-graph-digitizer.com, version 2.26.0.20). All virtual trial designs mimicked the observed clinical trials. Details about the clinical studies utilized for each compound can be found in the supplemental information (isavuconazole and posaconazole Tables S2 and S3, edoxaban Table S12, and isavuconazole Tables S17 and S18). A summary of the PK sampling data can be found in Table 2. In addition to the data listed in Table 2, the PK data from 16 DDI studies involving a total of 325 adult subjects were used for building and verification of the isavuconazole model. For posaconazole model development, additional PK data from six DDI studies involving 71 adult subjects were utilized for DDI model performance. Of note, due to the nature of extracting data from the literature, the numbers of PK samples for the posaconazole and voriconazole studies were estimated based on the number of subjects and the number of reported sampling times. All PK studies utilized in this analysis implemented an intensive PK sampling protocol.

**TABLE 2:** Pharmacokinetic sampling data utilized in PBPK model development

Compound	Formulation	Dose	Age (years)	N subjects	N samples
Isavuconazole	iv	40 mg single	23–45	6	169
		80 mg single	23–45	6	189
		160 mg single	23–45	6	170
		80–40 mg MD	26–45	6	378
		160–80 mg MD	26–45	6	408
	Oral	100 mg single	22–45	6	185
		200 mg single	22–45	6	204
		400 mg single	22–45	3	69
		50–100 mg MD	21–45	6	537
		100–200 mg MD	21–45	6	537
	iv	10 mg/kg MD	1 to < 6	9	123
			6 to < 12	8	105
			12 to < 18	7	95
	Oral		6 to < 12	9	114
			12 to < 18	8	110
Posaconazole^*a*^	iv	250 mg single	18–56	9	90
		300 mg single	18–56	9	81
		200 mg MD	49.1	21	294
		300 mg MD	52.4	24	336
	Oral suspension	100 mg single	19–45	16	496
		400 mg single	24–52	12	360
		400 mg single	22–43	8	160
	DR tablet	200 mg MD	33–59	10	200
		400 mg MD	31–56	9	180
	iv	3.5 mg/kg MD	2 to < 7	11	66
			7 to 17	19	114
		4.5 mg/kg MD	2 to < 7	18	108
			7 to 17	15	96
		6.0 mg/kg MD	2 to < 7	17	102
			7 to 17	24	144
	DR suspension	3.5 mg/kg MD	2 to < 7	5	30
			7 to 17	10	60
		4.5 mg/kg MD	2 to < 7	8	48
			7 to 17	8	48
		6.0 mg/kg MD	2 to < 7	7	102
			7 to 17	12	72
Voriconazole^*a*^	iv	5 mg/kg MD	2 to < 12	3	30
		7 mg/kg MD	2 to < 12	9	81
		8 mg/kg MD	2 to < 12	14	98

^*a*^ Number of collected samples was estimated based on the number of subjects and time points collected.

All reported simulated pharmacokinetic (PK) and statistical parameters were calculated by the simulator. Model performance was determined by calculating the GMFE (Eq. 1) and through the Guest criteria (Eq. 2) assuming 20% variability in pharmacokinetic parameters (19), where *δ* is a parameter that accounts for variability (1.25 for 20% variability), and *R_obs_* represents AUC_+inhibitor_/AUC_control_ ≥ 1 for inhibition DDIs. For induction DDIs, the reciprocal (AUC_control_/AUC_+inducer_) is used. The upper limit is calculated as R_obs_ * Limit, and the lower limit is calculated as *R_obs_/Limit,* where *R_obs_* is AUC_+inhibitor/inducer_/AUC_control_.


(1) $$GMFE=\exp\left(\frac{1}{n}\sum_{i=1}^{n}|ln\left(\frac{{Predicted}_{i}}{{Observed}_{i}}\right)|\right)$$

(2) $$Limit=\;\frac{\delta +2\left(R_{obs}-1\right)}{R_{obs}}$$

### Compound PBPK models

Specific details about the model building and verification of the isavuconazole PBPK model have been previously described (manuscript under peer review). Details on the model building and verification of the vincristine, posaconazole, and edoxaban PBPK compound models can be found in the supplemental information.

In short, the isavuconazole PBPK model was built using only the active moiety, isavuconazole, without the inactive prodrug, isavuconazonium sulfate. The equivalent dose of isavuconazole was administered (200 mg isavuconazole/372 mg isavuconazonium sulfate) for all simulations. The model was built using first-order absorption, full PBPK model for distribution, and elimination through CYP3A4/5 with biliary elimination. Extensive verification was performed for single-dose and multiple-dose administration in adults, and clinical DDI studies were used for inhibition and induction parameters. The model was translated to pediatric cancer patients and verified with multiple dose iv and oral data.

The posaconazole PBPK model was built based on the Simcyp-provided posaconazole model, literature data, and published PBPK model by Malik P, et al. (32). The Simcyp-provided posaconazole model was not deemed detailed enough (first-order metabolism, minimal PBPK model, and no P-gp inhibition) for the present application. Therefore, additional posaconazole model construction and verification was conducted. P-gp inhibition K_i_ value of 0.01 µmol/L was included in the model based on the *in vitro* study conducted by Lempers VJC et al. (12), where they found P-gp *in vitro* inhibition potential by isavuconazole and posaconazole to be the same. Verification of K_i,P-gp_ was performed using published DDI data with edoxaban and rivaroxaban (13). Model performance was verified using PK data in both adult and pediatric subjects with various posaconazole formulations, including suspension, DR tablet, and DR suspension under fasted and fed states.

The vincristine PBPK model was built based on the literature models published by Lee CM, et al. (33) and Reddy VP, et al. (34). Elimination through CYP3A4, CYP3A5, and P-gp was verified with published mass balance data (35) and *in vitro* metabolism data published by Dennison JB, et al. (36). Model performance was verified in both adults and pediatric subjects. Vincristine is dosed by BSA, with 1.5 mg/m^2^ administered as iv bolus with a maximum dose of 2 mg. Vincristine is formulated as a salt form as vincristine sulfate. The equivalent salt-corrected dose was 1.34 mg/m^2^ with a maximum dose of 1.79 mg.

To perform verification on the K_i,P-gp_ parameter used in both the isavuconazole and posaconazole models, an edoxaban PBPK model was constructed. Extensive details on the construction of this file can be found in the supplemental information. In short, the model was constructed based on the published PBPK model by Xu R, et al. and verified using published PK and DDI data (37).

The Simcyp simulator-provided PBPK model for voriconazole was determined to be sufficiently verified by Simcyp for the application of CYP3A4-mediated DDI prediction; however, voriconazole model model performance in a pediatric population was conducted using literature reported PK data (Table S8). The Simcyp simulator-provided PBPK models for cyclosporine, bupropion, dextromethorphan, digoxin, ethinyl estradiol, ketoconazole, midazolam, repaglinide, rifampicin, rivaroxaban, verapamil, and S-warfarin were also used without any modifications.

### Population models

The Simcyp simulator-provided PBPK population models for healthy volunteer population and cancer population were used without any modifications for most simulations. For the simulations in pediatric populations, the pediatric hematologic cancer population was used with minor modifications. Updates to the pediatric ontogeny for CYP3A4, CYP2C9, CYP2C19, and UGT1A4 were implemented in accordance with published data from Farhan N, et al. (32, 38) and Upreti and Wahlstrom (39). The equations used to determine the ontogeny distribution and the values utilized in these equations can be found in the supplemental information.

No ontogeny differences were included for P-gp transporter activity as literature reports that ABCB1 mRNA expression is completed relatively soon after birth. The expression was independent of age from 4 months to 20 years of age, with P-gp expression levels not found to be significantly different (40–42). Although the expression immediately after birth to <1 year was found to be lower than in adults, this difference does not impact the present analysis. In addition, due to the complexity of CYP3A5 expression, no specific ontogeny was included for CYP3A5. To simplify the approach to CYP3A5 abundance in EM populations, the CYP3A4-CYP3A5 correlation factor built within Simcyp was implemented. Based on literature data, CYP3A5 expression has been found to be correlated with CYP3A4 expression and abundance (43, 44). This correlation was implemented for adult simulations as well as pediatric simulations.

By default, the Simcyp populations utilized herein have a CYP3A5 PM phenotype frequency of 83%. This means that for a simulation conducted with *n* = 10 and trials = 10 (100 subjects), there will be ~17 subjects that are CYP3A5 expressors. Simulations with the default frequency give a general prediction for the overall population but do not provide more specific information on the exposure of an EM subject vs a PM subject. This distribution was used for all trials where the CYP3A5 phenotype was unknown. For predicted DDIs with vincristine, exclusively PM and EM populations were generated (i.e., 10 trials of 10 patients, all with either PM or EM CYP3A4 phenotype).

### Prediction of DDI between vincristine and azole antifungals

The verified models for isavuconazole, posaconazole, voriconazole, and vincristine were used to predict the magnitude of DDIs between isavuconazole, posaconazole, or voriconazole and vincristine in pediatric cancer patients. Simulations were conducted in the following age groups: 1 to <3 years old (isavuconazole only due to FDA-specific different dosage, e.g., 15 mg/kg), 1 to <6 years old, 6 to <12 years old, and 12 to <18 years old. In addition to age groups, simulations were conducted in populations that were either 100% CYP3A5 extensive metabolizers (EMs) or poor metabolizers (PMs).

The virtual population consisted of 10 subjects, 10 trials, and 50% female. Every simulation was 14 days long, with the triazole being administered for 10 days with a single iv bolus vincristine dose administered at the same time as the triazole on day 7. The vincristine dose was 1.5 mg/m^2^ (2 mg max) for every simulation. All triazoles were administered in their iv formulation to prevent confounding factors associated with oral absorption in cancer populations and to represent the largest possible magnitude of DDIs with the higher exposure of iv over oral administration. The following triazole dosing schemes were used based on clinical guidelines:

- Isavuconazole: 10 mg/kg (5.38 mg/kg isavuconazole equivalent dose, 200 mg max) Q8H for six doses, daily from days 3 to 10 via iv infusion over 1 h. For the 1 to <3-year-old cohort, 15 mg/kg (8.06 mg isavuconazole equivalent dose) was administered.
- Posaconazole: 6 mg/kg (300 mg max) Q12H for the first day, once daily from days 2 to 10, iv infusion over 1.5 h.
- Voriconazole: 9 mg/kg Q12H (over 3 h) for the first day, 8 mg/kg Q12H from days 2 to 10; iv infusion over 2.67 h.

### Sensitivity analysis

Sensitivity analysis was undertaken to evaluate values 10-fold higher and 10-fold lower than the value used in the isavuconazole and posaconazole models for K_i,CYP3A4_ (0.018–1.8 µmol/L), K_i,CYP3A5_ (0.018–1.8 µmol/L, isavuconazole only), and K_i,P-gp,liver_ (0.001–0.1 µmol/L). The f_u,mic_ for the posaconazole K_i,CYP3A4_ was not altered with the K_i_ value for that sensitivity analysis. Sensitivity analysis on voriconazole inhibition parameters was not conducted since extensive verification for this model has been previously performed by Simcyp. An additional sensitivity analysis was performed to evaluate values 10-fold higher and 10-fold lower than the value used in the final vincristine model for CL_int,P-gp,liver_ (0.45–45 µL/min/million cells), V_max,CYP3A4_ (0.15–14.9 pmol/min/pmol isoform), and V_max,CYP3A5_ (1.1–110 pmol/min/pmol isoform). For simplicity, these simulations were conducted using the DDI with posaconazole only since the magnitude in model sensitivity is expected to be similar across the different triazole compounds. The subject representative AUC ratio of vincristine when co-administered with isavuconazole or posaconazole was reported. The subject representative selected by the simulator was 3 years old and was a CYP3A5 PM, except for assessment of V_max,CYP3A5_ and K_i,CYP3A5,_ which the subject representative was CYP3A5 EM.

## Data Availability

Details for how researchers may request access to anonymized participant-level data, trial-level data, and protocols from Astellas-sponsored clinical trials can be found at https://www.clinicaltrials.astellas.com/transparency/.

## SUPPLEMENTAL MATERIAL

Supplemental material aac.00444-26-s0001.docx Tables S1 to S20; Fig. S1 to S7.

ASM does not own the copyrights to Supplemental Material that may be linked to, or accessed through, an article. The authors have granted ASM a non-exclusive, world-wide license to publish the Supplemental Material files. Please contact the corresponding author directly for reuse.
