# Supplementary material for: Physiologically based pharmacokinetic (PBPK) model to predict the magnitude of drug-drug interaction between vincristine and the azole antifungals isavuconazole, posaconazole, and voriconazole in children
Source: Antimicrob Agents Chemother. 2026 Jul 22;70(9):e00444-26. doi: 10.1128/aac.00444-26 (PMC13712836; doi:10.1128/aac.00444-26)
Supplement: Supplementary file 1 [file aac.00444-26-s0001.docx]

**Physiologically-based Pharmacokinetic (PBPK) Model to Predict the Magnitude of Drug-Drug Interaction Between Vincristine and the Azole Antifungals Isavuconazole, Posaconazole, and Voriconazole in Children**

Mary P Choules, PharmD, PhD, Amit Desai, PhD, Laura Lynn Kovanda, PhD, Yukio Otsuka, PhD, Roger Brüggemann, PhD, and Peter L Bonate, PhD

# Populations

The pediatric ontogeny followed the equations detailed in Eq. 1 and Eq. 2 where, *Adult_Max_* = fraction of maximum expression reached compared to adults, *F_Birth_* = fraction at birth, *Age_50_ =* age to half maximum, *n* = exponent, *Age* = post-natal age in years, and *Age-cap* = age at which the *Adult_Max_* is reached (1, 2). The ontogeny for CYP3A4 was changed from the default profile 1 in Simcyp (reported by Salem et al., (3, 4)) to profile 2 (reported by Upreti and Wahlstrom(5)). The only value that was altered from the default profile 2 was the *Age-cap^2^*, the value was increased from 12.5 to 25, which is the same as the Japanese pediatric population in Simcyp. Age-cap^2^ was included to stop the function from going below one. Increasing age-cap^2^ stops the function from going below 1 and prolongs the CYP3A4 elevations which is consistent from literature reports showing increased clearance of midazolam even past 16 years old (5). Further details about the two hepatic ontogenies within the Simcyp simulator can be found published by Codaccioni, et al. (6). In that publication, the preference for profile 2, where CYP3A4 abundance increases dramatically quickly after birth to levels above typical adult expression before decreasing to adult levels is explained. Modification to CYP2C9 and CYP2C19 were required for voriconazole simulations, and UGT1A4 was required for posaconazole simulations.

Eq. 1

$$\boldsymbol{Fraction of adult=}\left( \frac{\boldsymbol{Adult}_{\boldsymbol{Max}}\boldsymbol{-}\boldsymbol{F}_{\boldsymbol{Birth}}}{\boldsymbol{Age}_{\boldsymbol{50}}^{\boldsymbol{n}}\boldsymbol{+}\boldsymbol{Age}^{\boldsymbol{n}}} \right)\boldsymbol{\times}\boldsymbol{Age}^{\boldsymbol{n}}\boldsymbol{+}\boldsymbol{F}_{\boldsymbol{Birth}}$$

Eq. 2

$$\boldsymbol{Fraction}\mathbf{=}\boldsymbol{C}_{\mathbf{0}}\mathbf{+}\boldsymbol{C}_{\mathbf{1}}\boldsymbol{\cdot}\boldsymbol{exp}^{\boldsymbol{C}_{\mathbf{2}}\left( \boldsymbol{Age}\mathbf{-}{\boldsymbol{Age}\mathbf{-}\boldsymbol{cap}}^{\mathbf{1}} \right)}$$

Table S1.	Input parameters for pediatric population CYP ontogeny

| **Enzyme** | **Adult_max_** | **F_Birth_** | **Age_50_** | **n** | **Age-cap^1^** | **C_0_** | **C_1_** | **C_2_** | **Age-cap^2^** |
| --- | --- | --- | --- | --- | --- | --- | --- | --- | --- |
| **CYP3A4** | 1.7 | 0.15 | 0.1 | 1.3 | 2.5 | 0.7 | 1 | -0.1 | 25 |
| **CYP2C9** | 2.2 | 0.01 | 0.01 | 1.2 | 0.3 | 1 | 1.1 | -0.1 | 14 |
| **CYP2C19** | 2.3 | 0.16 | 0.3 | 2 | 0.8 | 1 | 1 | -0.5 | 12 |
| **UGT1A4** | 1.3 | 0.26 | 0.51 | 2.1 | 12 |  |  |  |  |

*Adult_Max_* = fraction of maximum expression reached compared to adults, *F_Birth_* = fraction at birth, *Age_50_ =* age to half maximum, *n* = exponent, *Age* = post-natal age in years, and *Age-cap* = age at which the *Adult_Max_* is reached

# Posaconazole

The PBPK model for posaconazole was constructed based on previously reported PBPK models provided by Simcyp and published in the literature (1, 7, 8) . The clinical data utilized in the model building and verification of the posaconazole PBPK model are listed in Table S2. Clinical DDI data utilized to verify posaconazole CYP3A4 and P-gp inhibition is listed in Table S3. A summary of the final posaconazole PBPK model parameters are listed in Table S4.

The model was first built using healthy volunteer data then adjusted for patients before final translation to pediatric cancer patients. First, the model was built using single dose IV data in healthy volunteers from Kersemaekers, et. al. (9) and fasted and fed suspension data from Krishna, et. al. (10). Contribution of metabolism through UGT1A4 was maintained from the Simcyp verified compound file; however, the compound file was updated to ADAM model for absorption and biliary elimination which required additional verification of the elimination pathways. Elimination pathways were verified using mass balance data from Krieter, et. al.(11). Verification of the oral suspension formation was performed using 400 mg oral suspension in fasted and fed conditions from Krishna, et. al.(12). Delated release (DR) tablet formulation was built using dissolution data from Pandav, et. al.(13), no other parameters were changed between the suspension and DR tablet formations. The DR tablet formation was verified with multiple dose 200 mg and 400 mg dose data from Krishna, et. al.(14). When adopting the model for patients, literature data reports that V_ss_ and CL were found to be >2-fold in critically ill patients over healthy volunteers. Therefore, the V_ss_ and active hepatic scaler in the model was updated to 7.77 L/kg and 14.8, respectively. These were optimized using data from multiple dose 200 mg iv data and verified with 400 mg iv data. The final adult model PK parameters compared to observed data can be found in Table S5. Visual fits can be found in Figure S1, Figure S2, and Figure S3. Of note, posaconazole is highly peripherally distributed and peripheral sampling was conducted via peripheral iv in all studies; therefore, peripheral sampling was reported for all posaconazole simulations.

The model was built using ADAM model for absorption, full PBPK for distribution calculated using method 1 (Poulin-Theil/Berezhkovskiy), enzymatic elimination through UGT1A4, and biliary elimination with enterohepatic recirculation (EHRC). P_eff,man_ was predicted using the Mechanistic Passive Regional Permeability Predictor (MechPeff) model using calculation through method 1 (P_trans,0_ = a·P_o:w_^b^). Fraction unbound in the gut (f_uGut_) was assumed to be the same as f_u_, based on literature posaconazole models. Bile micelle mediated solubilization enhancement was predicted by the Simcyp simulator and verified with fasted suspension data. For the food effect seen with the posaconazole suspension, in vitro studies reported improved solubility of posaconazole in intestinal fluids containing high phospholipids and free fatty acids(15). To capture this effect, a fatty acid solubilization constant (K_FA_) was included in the model. The values calculated using the study data by Silva, et. al., did not capture the observed food effect; therefore, the parameter was optimization was required.

To verify CYP3A4 and P-gp inhibition by posaconazole, several DDI simulations were carried out. A summary of these simulations compared to observed data can be found in Figure S4 and Table S6. In the posaconazole PBPK file provided by Simcyp, an inhibition parameter for P-gp was not included; therefore, a value for K_i,P-gp_ had to be identified. In an in vitro study conducted by Lempers, et. al. (16), explored the inhibition potential of all marketed triazole compounds against several common drug transporters, including P-gp. The IC_50_ for posaconazole against P-gp was determined to be 3 µM (95% CI: 2.2-4.1 µM), which was the same as isavuconazole (3µM, 95% CI: 2.7-3.9 µM). During isavuconazole compound development, an in vitro K_i,P-gp_ was optimized to 0.01 µM based on a clinical DDI study with digoxin. Therefore, as a base value, posaconazole K_i,P-gp_ was set to 0.01 µM. To verify K_i,P-gp_, simulations were carried out with rivaroxaban and edoxaban as P-gp substates (see edoxaban section for compound development and verification). The observed geometric mean (GM) ratio for both studies was captured by the PBPK model without modification to K_i,P-gp_. Therefore, no further modification was conducted for this parameter. However, due to the uncertainty of this parameter and the impact to the predicted ddi potential with vincristine, a sensitivity analysis was conducted (Table S19). Conclusions from this sensitivity analysis are discussed in the main manuscript body.

The posaconazole PBPK model optimized for adult cancer patients was then translated to pediatric cancer patients with no further compound model changes. The pediatric cancer population and the ontogeny used for UGT1A4 are discussed in the main manuscript body. Comparison of observed and predicted PK parameters after multiple dose iv and DR suspension administration of posaconazole can be found in Table S7. Model performance in pediatric cancer patients was quite good, with a GMFE of 1.15-1.16 across AUC, C_max_, and C_min_. This final model was then utilized for the predictive DDI simulations with vincristine.

Table S2.	Clinical data utilized in the posaconazole compound file development

| **Study (reference)** | **Dose** | **Form** | **Cohort** | **N** | **Age**  **(years)** | **BMI**  **(kg/m^2^)** |
| --- | --- | --- | --- | --- | --- | --- |
| Adult (9) | 250, 300 mg  30 min infusion | iv | Healthy  (0.54 female) | 9 | 18-56 | 19-35 |
| Adult (10) | 100 mg fasted or fed | Oral suspension | Healthy  (0.50 female) | 16 | 19-45 | 21.3-30.5 |
| Adult food effect (12) | 400 mg fasted or fed | Oral suspension | Healthy  (0.50 female) | 12 | 24-52 | 23-29.3 |
| Adult mass balance (11) | 400 mg high fat fed | Oral suspension | Healthy males | 8 | 22-43 | 22.6-25 |
| Adult (14) | 200 mg day 1, Q12H day 6, QD day 7-14 | DR tablet | Healthy  (0.5 female) | 10 | 33-59 | 61-100* |
| Adult (14) | 400 mg day 1, Q12H day 6, QD day 7-14 | DR tablet | Healthy  (0.33 female) | 9 | 31-56 | 61-86* |
| Adult (17) | 200 mg-1.5 h infusion  day 1: Q12H  day 2-14: daily | iv | Cancer patients  (0.38 female) | 21 | 49.1 (14.7) | 77.2 (11.4)* |
| Adult (17) | 300 mg-1.5 h infusion  day 1: Q12H  day 2-14: daily | iv | Cancer patients  (0.46 female) | 24 | 52.4 (13.4) | 76.9 (13.8)* |
| Pediatric(18) | 3.5 mg/kg (300 max)  day 1: Q12H  day 2-10: daily | iv or DR suspension | Children with neutropenia | 35 | 2-17 | NR |
| Pediatric(18) | 4.5 mg/kg (300 max)  day 1: Q12H  day 2-10: daily | iv or DR suspension | Children with neutropenia | 31 | 2-16 | NR |
| Pediatric(18) | 6 mg/kg (300 max)  day 1: Q12H  day 2-10: daily | iv or DR suspension | Children with neutropenia | 49 | 2-17 | NR |

*weight (kg) reported

SAD: single ascending dose, NR: not reported, MAD: multiple ascending dose, QXH: every X hours

Table S3.	Summary of DDI studies used in the development and verification of the posaconazole PBPK model

| **Posaconazole Dosing** | **Victim** | **Victim Dosing** | **Cohort** | **N** | **Age**  **(yr)** | **Ref** |
| --- | --- | --- | --- | --- | --- | --- |
| 50 mg daily susp  day 1-7, 9-10: high fat meal, day 8: fasted | midazolam | day 8: 2 mg oral | Healthy  (female 0.27) | 11 | 18-45 | (19) |
| 100 mg daily susp  day 1-7, 9-10: high fat meal, day 8: fasted | midazolam | day 8: 2 mg oral | Healthy  (female 0.25) | 12 | 19-55 | (19) |
| 200 mg daily susp  day 1-7, 9-10: high fat meal, day 8: fasted | midazolam | day 8: 2 mg oral | Healthy  (female 0.50) | 12 | 21-51 | (19) |
| Q12H suspension  day 1-7: 200 mg  day 8-14: 400 mg | midazolam | day 6/13:  2 mg oral  day 7/14: 0.4 mg iv over 30 min | Healthy (female 0.083) | 12 | 28-53 | (20) |
| day 1: 300 mg Q12H DR tablet | rivaroxaban | day 1: 25 µg 1 hr after | Healthy  (0.42 female) | 12 | 20-48 | (21) |
| day 1: 300 mg Q12H DR tablet | edoxaban | day 1: 50 µg 1 hr after | Healthy  (0.42 female) | 12 | 20-48 | (21) |

susp: suspension, iv: intravenous, DR: delayed release, yr: year, QXH: every X hours, QD: every day

Table S4	Input parameters for the posaconazole PBPK model

| **Sample parameter** | **Value** | | **Assumption(s) and ref(s)** |
| --- | --- | --- | --- |
| **Physicochemical properties and blood binding** | | | |
| **Compound type** | Diprotic base | |  |
| **Molecular weight (g/mol)** | 700.8 | | (7) |
| **Log P** | 4.6 | | (7) |
| **pKa 1** | 4.6 | | (7) |
| **pKa 2** | 3.6 | | (7) |
| **Polar surface area (Å²)** | 111.79 | | Computed |
| **Hydrogen bond donors** | 1 | | (22) |
| **Hydrogen bond acceptors** | 11 | | (22) |
| **B/P ratio** | 1.145 | | Simcyp predicted |
| **f_u_** | 0.02 | | (7) |
| **Plasma binding protein** | HSA | | (23) |
| **Absorption** | | | |
| **Absorption model** | ADAM | |  |
| **UBL fluid volumes** | Yes | |  |
| **f_u,gut_** | 0.02 | | Assumed same as plasma |
| **P_eff,man_ (10^-4^ cm/s)** | 13.31 | | Simcyp predicted Mech P_eff_ model method 1, total concentration (bound+unbound) |
| **P_trans,0_ (10^-6^ cm/s)** | 1953 | |  |
| **Intrinsic Solubility (mg/mL)** | 9.8·10^-4^ | | (7) |
| **Solubility Factor (SF1)** | 71 | | (7) |
| **Bile Micelle Mediated Sol.** | Yes | |  |
| **Bile micelle: buffer partition coefficients** | | |  |
| **logK_m:w_ Neutral** | 5.694 | | Simcyp predicted |
| **logK_m:w_ Ion** | 3.694 | | Simcyp predicted |
| **K_FA_ Neutral (mM^-1^)** | 0.98 | | optimized |
| **Fraction API Diss. (%)**  (suspension only) | 2.3 | | (7) |
| **DR tablet (CR) parameters** | | | |
| **pH Threshold** | 6.8 | | (13) |
| **Dissolution Profile**  **Piecewise cubic polynomial**  **(both fasted and fed states)** | **Time (h)** | **Diss. (%)** |  |
|  | 0.17 | 84.69 |  |
|  | 0.25 | 87.65 |  |
|  | 0.33 | 90.31 |  |
|  | 0.5 | 90.58 |  |
|  | 0.75 | 92.61 |  |
| **Segregated Transit Time** | fine particles = fluid | | |
| **Diffusion Layer Model** | Particle Population Balance | |  |
| **Particle h_eff_ Model** | Hintz-Johnson | |  |
| **Particle Size Distribution** | Monodispersed | |  |
| **Radius (µm)** | 10 | | (7) |
| **Density (g/mL)** | 1.0 | | Posaconazole MSD |
| **Critical Supersaturation Ratio** | 2.9 | | (7) |
| **Precipitation rate constant (PRC) (h^-1^)** | 4.0 | | Default value |
| **Secondary PRC (h^-1^)** | 7.28 | | (7) |
| **Distribution** | | | |
| **Distribution model** | Full PBPK | |  |
| **V_ss_ (L/kg)** | 3.63 (healthy)  7.77 (patients) | | Predicted by Method 1 |
| **Kp scalar** | 0.32 (healthy)  0.77 (patients) | | Optimized average V_z_ from IV (healthy -3.61 L/kg (24), patients – 7.77 L/kg (25) |
| **Tissue:Plasma Partition Coefficients** |  | |  |
| **Brain** | 2.45 | | Calculated from mean human tissue concentrations (26) |
| **Heart** | 7.2 | |  |
| **Kidney** | 12.4 | |  |
| **Liver** | 20.8 | |  |
| **Lung** | 9.2 | |  |
| **Elimination** | | | |
| **UGT1A4** |  | |  |
| **V_max_ (pmol/min/mg microsomal protein)** | 16.9 | | (8) |
| **K_m_ (µM)** | 15.9 | |  |
| **f_u,mic_** | 0.175 | | Simcyp calculated |
| **Active Hepatic Scalar** | 10.5 (healthy)  14.8 (patients) | | Optimized to CL_IV_ (healthy - 6.68 L/h (24), patients- 16.8 L/h (25) |
| **CL_int_ Bile (µL/min/10^6^)** | 3.78 | | Retrograde calculated using 66% f_CL,Bile_ and CL_IV_ 6.68 L/h |
| **% available for reabsorption** | 76 | | Estimated to recover unchanged posaconazole in feces |
| **CL_R_ (L/h)** | 0.0114 | | (11) |
| **Interaction** | | | |
| **K_i_ CYP3A4 (µM) (fu_mic_)** | 0.185 (0.514) | | Simcyp posaconazole summary sheet |
| **K_i_ ABCB1/P-gp (µM)** | 0.01 | | Optimized based on literature (21, 27) |

LogP: octanol:buffer partition coefficient, B/P ratio: blood-to-plasma partition ratio, fu: fraction unbound, HSA: Human Serum Albumin, ADAM: advanced dissolution, absorption and metabolism model, UBL: Unstirred Boundary Layer, P_eff,man_: Human jejunum effective permeability, P_trans,0_: membrane passive intrinsic transcellular permeability, Mech P_eff_: mechanistic passive reginal permeability, logK_m:w_: logarithm of bile micelle: buffer partition coefficient, K_FA_: fatty acid solubilization constant, DR: delayed release, CR: controlled release, V_ss_: volume of distribution at stead-state, K_p_ scalar: value applied to all tissue:plasma partition coefficients, V_max_: maximum rate of metabolism, K_m_: Michaelis constant, f_u,mic_: fraction of unbound drug in the in vitro microsomal incubation, CL_R_: renal clearance, CL_int_: intrinsic clearance, K_i_: concentration of inhibitor that supports half maximal inhibition

Table S5	Predicted and observed PK parameters for single and multiple dose posaconazole in adults

|  | **AUCinf (ng·h/mL)** | | | **Cmax (ng/mL)** | | | **CL (L/h)** | |
| --- | --- | --- | --- | --- | --- | --- | --- | --- |
|  | **Predicted** | **Observed** | | **Predicted** | **Observed** | | **Predicted** | **Observed** |
| **250 mg IV** (9) | 41,600  (33) | 41,500  (41) | | 2,200  (15) | 2,260  (26) | | 6.68  (34) | 6.68  (29) |
| **300 mg IV** (9) | 50,200  (33) | 46,400  (26) | | 2,640  (15) | 2,840  (30) | | 6.66  (34) | 6.90  (27) |
| **100 mg oral suspension fasted** (10) | 3,910  (51) | 3,420  (44) | | 101  (35) | 84.0  (62) | | 31.7  (47) | 34.0  (38) |
| **100 mg oral suspension fed** (10) | 11,200  (46) | 8,750  (24) | | 270  (30) | 243  (18) | | 10.8  (44) | 12.1  (26) |
| **400 mg oral suspension fasted** (12) | 5,140  (53) | 4,280  (44)* | | 131  (39) | 181  (106) | | 99.3  (50) | 110  (62) |
| **400 mg oral suspension fed** (12) | 26,200  (46) | 20,900  (49)* | | 607  (36) | 555  (44) | | 18.5  (44) | 24.3  (58) |
| **400 mg oral suspension fed (MB study)** (11) | 27,300  (52) | 26,100  (26) | | 623  (39) | 654  (19) | | 18.6  (51) | 16.3  (30) |
| **200 mg DR tab, Day 1** (14) | 23,700  (39)* | 23,000  (23)* | | 831  (27) | 778  (29) | | 9.76  (40) | 8.80  (26) |
| **200 mg DR tab, Day 14** (14) | 28,300  (43)† | 31,400  (32)† | | 1,690  (36) | 1,800  (31) | | 8.46  (0.44) | NR |
| **400 mg DR tab, Day 1** (14) | 51,100  (35)* | 42,800  (35)* | | 1,760  (23) | 1,290  (29) | | 8.88  (40) | 9.55  (34) |
| **400 mg DR tab, Day 14** (14) | 63,900  (40)† | 56,600  (54)† | | 3,720  (32) | 2,940  (46) | | 7.35  (43) | NR |
| **200 mg IV, Day 1**  (17) | 5,740  (17)† | 5,390  (29)† | | 960  (15) | 990  (47) | | 35.9  (17) | NR |
| **200 mg IV, Day 14**  (17) | 29,800  (35)† | 28,200  (51)† | | 1,930  (25) | 1,950  (50) | | 7.57  (38) | NR |
| **300 mg IV, Day 1**  (17) | 9,370  (18)† | 8,240  (26)† | | 2,190  (17) | 1,590  (61) | | 33.0  (18) | NR |
| **300 mg IV, Day 14**  (17) | 47,100  (36) | 34,300  (42)† | | 3,010  (26) | 2,610  (39) | | 7.25  (39) | NR |
| **GMFE** | **1.14** | | **1.14** | | | **1.11** | | |

*AUC_last_

†AUC_tau_

iv: intravenous, MB: mass balance, DR tab: delayed release tablet, NR: not reported, GMFE: geometric mean fold error, AUC_inf_: area under the curve from 0 to infinity, Cmax maximum concentration, CL: total clearance (dose/AUC_inf_)

CL/F reported for oral formulations

data presented as arithmetic mean (%coefficient of variance) using peripheral sampling

Figure S1	Predicted and observed peripheral plasma concentration curve of posaconazole in adult healthy volunteers receiving posaconazole suspension 400 mg in fasted or fed state

**Fasted	Fed**

Observed data extracted from Krishna, et. al. (12)

Black circles: observed clinical data, solid black line: simulated arithmetic mean, dark grey line: simulated 95th percentile, light grey line: simulated 5th percentile

Figure S2	Predicted and observed peripheral plasma concentration curve of posaconazole in adult healthy volunteers receiving posaconazole DR tablet 200 or 400 mg

**200 mg	400 mg**

Observed data extracted from Krishna, et. al.(14)

Black circles: observed clinical data, solid black line: simulated arithmetic mean, dark grey line: simulated 95th percentile, light grey line: simulated 5th percentile

Figure S3	Predicted and observed peripheral plasma concentration curve of posaconazole in adult cancer patients receiving multiple dose posaconazole iv 200 mg or 300 mg

**200 mg	300 mg**

Observed data extracted from Maerten, et. al.(17)

Black circles: observed clinical data, solid black line: simulated arithmetic mean, dark grey line: simulated 95th percentile, light grey line: simulated 5th percentile

Figure S4	Visual PBPK model performance assessment for posaconazole DDI studies

grey squares: predicted/observed (P/O) AUC or C_max_ ratio of study data, solid black line: P/O = 1, dotted lines: GUEST criteria with 20% variability(28), dash lines: upper and lower 2-fold threshold

Table S6.	Summary of predicted by the PBPK model and observed PK parameters (AUC and C_max_ ratio) of DDI for posaconazole as the perpetrator

|  | **Without Posaconazole** | | **With Posaconazole** | |  | |
| --- | --- | --- | --- | --- | --- | --- |
|  | **AUC_inf_ (ng*h/mL)** | **C_max_**  **(ng/mL)** | **AUC_inf_ (ng*h/mL)** | **C_max_**  **(ng/mL)** | **AUC_inf_ ratio** | **C_max_ Ratio** |
| **50 mg posaconazole - 2 mg oral midazolam** (19) | | | | | | |
| **Observed** | 24 | 9.33 | 73 | 18.3 | **3.04** | **1.96** |
| **90% CI** |  |  |  |  | (2.57, 3.60) | (1.65, 2.32) |
| **Predicted** | 19.0 | 5.56 | 49.3 | 11.5 | **2.59** | **2.07** |
| **90% CI** |  |  |  |  | (2.46, 2.74) | (1.98, 2.17) |
| **Criteria** |  |  |  |  | **1.73 - 5.33** | **1.21 - 3.17** |
| **100 mg posaconazole - 2 mg oral midazolam** (19) | | | | | | |
| **Observed** | 28.6 | 9.18 | 113 | 21.9 | **3.95** | **2.38** |
| **90% CI** |  |  |  |  | (3.05, 5.11) | (1.96, 2.89) |
| **Predicted** | 19.7 | 5.69 | 66.7 | 13.4 | **3.38** | **2.36** |
| **90% CI** |  |  |  |  | (3.18, 3.60) | (2.24, 2.48) |
| **Criteria** |  |  |  |  | **2.18 - 7.15** | **1.41 - 4.01** |
| **200 mg posaconazole - 2 mg oral midazolam** (19) | | | | | | |
| **Observed** | 22.6 | 9.26 | 135 | 24.9 | **6.00** | **2.69** |
| **90% CI** |  |  |  |  | (5.15, 6.98) | (2.46, 2.93) |
| **Predicted** | 19.8 | 5.87 | 83.5 | 15.4 | **4.23** | **2.62** |
| **90% CI** |  |  |  |  | (3.92, 4.55) | (2.48, 2.77) |
| **Criteria** |  |  |  |  | **3.20 - 11.3** | **1.56 - 4.63** |
| **200 mg Q12H posaconazole - 2 mg oral midazolam** (20) | | | | | | |
| **Observed** | 30.9* | 7.0 | 137* | 15.4 | **4.59*** | **2.26** |
| **90% CI** |  |  |  |  | (4.12, 5.11) | (2.02, 2.53) |
| **Predicted** | 26.8* | 4.54 | 141* | 13.9 | **5.24*** | **3.06** |
| **90% CI** |  |  |  |  | (4.68, 5.86) | (2.82, 3.32) |
| **Criteria** |  |  |  |  | **2.50 - 8.43** | **1.35 - 3.77** |
| **200 mg Q12H posaconazole - 0.4 mg iv midazolam** (20) | | | | | | |
| **Observed** | 10.8* | 5.6 | 52* | 7.3 | **4.62*** | **1.30** |
| **90% CI** |  |  |  |  | (4.02, 5.30) | (1.13, 1.48) |
| **Predicted** | 17.2* | 6.95 | 66.4* | 10.1 | **3.87*** | **1.45** |
| **90% CI** |  |  |  |  | (3.41, 4.38) | (1.38, 1.53) |
| **Criteria** |  |  |  |  | **2.51 - 8.49** | **0.914 - 1.85** |
| **400 mg Q12H posaconazole - 2 mg oral midazolam** (20) | | | | | | |
| **Observed** | 30.9* | 7.00 | 148* | 16.3 | **4.97*** | **2.38** |
| **90% CI** |  |  |  |  | (4.46, 5.54) | (2.13, 2.66) |
| **Predicted** | 26.8* | 4.54 | 162* | 14.9 | **6.05*** | **3.29** |
| **90% CI** |  |  |  |  | (5.34, 6.85) | (3.01, 3.59) |
| **Criteria** |  |  |  |  | **2.69 - 9.19** | **1.41 - 4.01** |
| **400 mg Q12H posaconazole - 0.4 mg iv midazolam** (20) | | | | | | |
| **Observed** | 10.8* | 5.6 | 70.2* | 9.4 | **6.24*** | **1.62** |
| **90% CI** |  |  |  |  | (5.43, 7.16) | (1.41, 1.86) |
| **Predicted** | 17.2* | 6.95 | 82.3* | 11.0 | **4.79*** | **1.58** |
| **90% CI** |  |  |  |  | (4.15, 5.52) | (1.49, 1.68) |
| **Criteria** |  |  |  |  | **3.32 - 11.7** | **1.05 - 2.49** |
| **300 mg Q12H posaconazole - 25 µg rivaroxaban**(21) | | | | | | |
| **Observed** | 3.63 | 0.822 | 4.98 | 1.15 | **1.38** | **1.39** |
| **90% CI** |  |  |  |  | NR | NR |
| **Predicted** | 2.17 | 0.269 | 3.08 | 0.309 | **1.42** | **1.15** |
| **90% CI** |  |  |  |  | (1.35, 1.50) | (1.12, 1.17) |
| **Criteria** |  |  |  |  | **0.947 - 2.01** | **0.952 - 2.03** |
| **300 mg Q12H posaconazole - 50 µg edoxaban** (21) | | | | | | |
| **Observed** | 0.862 | 0.132 | 1.80 | 0.284 | **2.09** | **2.15** |
| **90% CI** |  |  |  |  | NR | NR |
| **Predicted** | 1.63 | 0.255 | 3.23 | 0.446 | **1.98** | **1.75** |
| **90% CI** |  |  |  |  | (1.91, 2.05) | (1.66, 1.84) |
| **Criteria** |  |  |  |  | **1.27 - 3.43** | **1.30 - 3.55** |

*AUC_last_ reported: area under the curve from 0 to last measurable point

AUC_inf_: area under the curve from 0 to infinity, C_max_: maximum concentration, CI: confidence interval, Q12H: every 12 hours, iv: intravenous, AUC ratio: AUC(with inhibitor)/AUC(without inhibitor), C_max_ ratio: C_max_(with inhibitor)/C_max_(without inhibitor)

Criteria determined by GUEST method assuming 20% variability in pharmacokinetic parameters(28)

Table S7	Predicted and observed PK parameters for multiple dose posaconazole in pediatric patients at day 10 of dosing

|  | **AUC_24_ (ng·h/mL)** | | | **C_max_ (ng/mL)** | | | **C_min_ (ng/mL)** | |
| --- | --- | --- | --- | --- | --- | --- | --- | --- |
|  | **Predicted** | **Observed** | | **Predicted** | **Observed** | | **Predicted** | **Observed** |
| **2-7 years old** | | | | | | | | |
| **3.5 mg/kg iv** | 17,000 (30.6) | 17,800 (55.0) | | 1,770 (14.9) | 1,590 (43.1) | | 415 (47.9) | 400 (81.3) |
| **4.5 mg/kg iv** | 22,300 (30.1) | 25,600 (39.8) | | 2,230 (15.1) | 2,320 (39.8) | | 552 (46.4) | 501 (56.8) |
| **6 mg/kg iv** | 30,700 (31.2) | 31,100 (48.9) | | 3,120 (15.6) | 3,060 (54.1) | | 769 (47.8) | 626 (104.8) |
| **3.5 mg/kg DR suspension** | 12,500 (35.7) | 12,200 (36.0) | | 834 (27.1) | 884 (44.4) | | 326 (51.8) | 254 (45.6) |
| **4.5 mg/kg DR suspension** | 17,200 (35.5) | 21,600 (64.5) | | 1,130 (27.0) | 1,550 (40.8) | | 462 (50.8) | 476 (165) |
| **6 mg/kg DR suspension** | 23,400 (35.4) | 23,000 (47.3) | | 1,520 (26.6) | 1,510 (43.4) | | 626 (51.3) | 542 (68.8) |
| **7 to 17 years old** | | | | | | | | |
| **3.5 mg/kg iv** | 21,300 (30.9) | 27,300 (49.7) | | 1,990 (17.3) | 2,450 (72.7) | | 574 (45.3) | 670 (65.1) |
| **4.5 mg/kg iv** | 25,000 (30.8) | 29,800 (42.9) | | 2,410 (16.3) | 2,310 (40.3) | | 646 (47.1) | 737 (66.0) |
| **6 mg/kg iv** | 33,800 (31.4) | 44,200 (41.5) | | 3,140 (18.6) | 3,340 (39.4) | | 890 (45.7) | 1160 (60.4) |
| **3.5 mg/kg DR suspension** | 18,000 (33.8) | 20,700 (33.8) | | 1,420 (22.9) | 1,340 (30.8) | | 504 (43.8) | 579 (44.9) |
| **4.5 mg/kg DR suspension** | 19,500 (28700) | 28,700 (33.7) | | 1,300 (32.6) | 1,670 (28.5) | | 539 (51.5) | 790 (48.2) |
| **6 mg/kg DR suspension** | 27,700 (32.1) | 25,000 (184.3) | | 2,180 (22.6) | 1,370 (178.5) | | 743 (46.0) | 713 (300.6) |
| **GMFE** | **1.16** | | **1.15** | | | **1.16** | | |

Observed data from Groll AH, et. al. (29)

iv: intravenous, DR: delayed release, GMFE: geometric mean fold error, AUC_24_: area under the curve from 0 to 24h, C_max_: maximum concentration, C_min_: minimum concentration

data presented as geometric mean (% geometric coefficient of variance)

# Voriconazole

The PBPK model for voriconazole provided by Simcyp was verified extensively in adult populations by Simcyp. Therefore, the compound file was only tested in pediatric populations to ensure that exposure of voriconazole in the virtual pediatric populations could capture the observed values. Observed data was extracted from two different studies, one by Michael, et. al.(30) and a second from Mori, et. al.(31). The observed versus predicted PK parameters are summarized in Table S8. Although exposure of voriconazole in pediatric populations is reported to be highly variable, the performance of the voriconazole PBPK model in pediatric patients was determined to be sufficient for use in predicting DDI with vincristine.

Table S8	Predicted and observed PK parameters for multiple dose voriconazole in pediatric patients

|  | **AUC_0-12h_ (ng·h/mL)** | | **C_max_ (ng/mL)** | | **C_min_(ng/mL)*** | |
| --- | --- | --- | --- | --- | --- | --- |
|  | **Predicted** | **Observed** | **Predicted** | **Observed** | **Predicted** | **Observed** |
| **2 to <12 years** | | | | | | |
| **8 mg/kg iv (day 7)**(31) | 66,800 (53.8) | 51,900 (51) | 8,155 (42.2) | 7,750 (38) | 3,810 (460-1700) | 2,530 (596-9360) |
| **7 mg/kg iv (day 3)**(30) | 42,200 (40.1) | 49,300 (69.1) | 7,480 (33.2) | 11,400 (49.7) | 1,870 (305-7400) | 2,160 (0-16300)† |
| **5 mg/kg iv (day 3)**(30) | 25,900 (39.8) | 26,100 (57.5) | 4,950 (28.8) | 5,830 (136) | 1,100 (195-3630) | 930 (400-3000)† |
| **GMFE** | **1.15** | | **1.24** | | **1.27** | |

*Geometric mean (range: minimum – maximum) reported for C_min_

†Reported over 10 days of therapy

iv: intravenous, GMFE: geometric mean fold error, AUC_0-12h_: area under the curve from 0 to 12h, C_max_: maximum, C_min_: minimum concentration/trough concentration

data presented as geometric mean (% geometric coefficient of variance)

# Vincristine

Vincristine is a vinca alkaloid chemotherapy drug which disrupts cancer cell division by inhibiting the formation of microtubules which prevents the mitosis phase of the cell cycle and ultimately leading to cell death(32). Vincristine is typically administered iv bolus with dosing calculated by body surface area (BSA) at a dose of 1.5 mg/m^2^ with a maximum dose of 2 mg(32) . Vincristine is primarily metabolized by CYP3A4/5, with greater affinity for CYP3A5(33, 34). Excretion is primarily in the feces through the bile via P-glycoprotein (P-gp), with 69% of radioactivity recovered in the feces after 72 hours(35). There was considerably less radioactivity excreted in the urine (12%). It was estimated that approximately 34% of the administered dose was eliminated as metabolites.

The PBPK model for vincristine was constructed based on previously reported PBPK models (36, 37). The clinical data utilized in the model building and verification of the vincristine PBPK model are listed in Table S9. The model was first built and verified using adult observed PK data then verified using observed PK in children/pediatric subjects. The final parameters used in the pediatric vincristine PBPK model can be found in Table S10. Most initial model parameters were obtained from Reddy et al. A summary of PK parameters predicted by the PBPK models and reported from observed data can be found in Table S11. A visual fit of the pediatric data set from Barnett, et al can be found in Figure S5.

The vincristine model was built using the ADAM absorption model with Mech P_eff_ model 1 prediction, full PBPK model for distribution and permeability limited liver model for P-gp transport with EHRC. Although vincristine is administered by iv only, the ADAM absorption model was utilized to apply EHRC.

For initial simulations, the value reported in the literature from pediatric population was used for V_ss_ (15.9 L/kg)(38). A large K_p_ scaler of 82 was required to capture the observed V_dss_, most likely from vincristine’s high binding affinity in tissues which may not be fully captured by equations used to calculate and predict V_ss_ through physiochemical properties alone. The V_ss_ utilized in the PBPK model was verified in an adult and pediatric populations and captured the observed V_d_ of 12.8 L/kg (39) (simulated = 12.8 L/h) in adults. Interestingly, the variability in reported V_d_ was larger in pediatric cancer patients than adult cancer patients, with reported %CV of 80% (range: 2.76-51.34 L/kg, n = 52)(38). The PBPK model simulated V_ss_ in the pediatric cancer population was 11.5 L/h (%CV: 16.1%), slightly lower than reported. However, considering the large variability and unpredictability of vincristine’s V_ss_ in pediatric subjects, the value remained unchanged in the final pediatric model.

A mass balance study conducted in 4 non-Hodgkin’s lymphoma adult patients receiving a 2 mg equivalent dose of [^3^H]-vincristine sulfate, reported 33% and 69% radioactivity recovered in the feces by 24 hours and 72 hours, respectively (35) . After 72 hours, 12% radioactivity was excreted in the urine. The analysis reported that approximately 34% of the administered vincristine dose was excreted as metabolites (40% and 46% feces and urine, respectively). Total recovery after 72 hours was estimated to be 81%. Based on these reported values, approximately 41% and 6.5% of unchanged vincristine was recovered in the feces and urine, respectively. After a 72-hour simulation using the vincristine PBPK model, 80% of the administered dose was recovered, 33% through hepatic and gut metabolism, 42% unchanged vincristine in the feces, and 6% in the urine. The percentage available for reabsorption (19.8%) was optimized based on the calculated unchanged vincristine present in the feces after 72 hours.

Vincristine metabolism was found to be more efficient through CYP3A5 than through CYP3A4, with intrinsic clearance of vincristine through CYP3A5 estimated to be ~12-fold higher than by CYP3A4 (34). Vincristine affinity was found to be similar between CYP3A4 and CYP3A5. Formation of M1 was stimulated ~3-fold by the presence of cytochrome b_5_, but relative efficiencies of M1 formation when compared to the absence of cytochrome b_5_ were unaffected. Considering that the addition of cytochrome b_5_ leads to a more physiologically relevant result, the V_max_ from exogenous and coexpressed b_5_ was explored using the PBPK model. The values from exogenous b_5_ (3:1 ratio) captured the mass balance data (35) and the PK was consistent with reported half-life difference between CYP3A5 extensive metabolizers (EM) and poor metabolizers (PM)(36). Although PK parameters for PM vs EM were not reported by Reddy, et. al, the authors reported that the half-life of PM vs EM was 79 h (range 64-96 h) and 18 h (range 11-25 h), respectively. The present PBPK model predicted a half-life of 42.6 (17.8-79.5 h) and 18.6 h (9.4-26.2 h) in PM and EM populations, respectively. It was unclear why the half-life reported by Reddy, et al. was larger than the present PBPK model considering the good the visual fit of the PBPK model (Figure S5). However, considering that the majority (83%) of subjects are expected to be PM through CYP3A5, and the model fit for other adult and pediatric datasets were acceptable (AUC GMFE 1.02), the model was considered well verified for the intended purpose.

Table S9.	Clinical data utilized in the vincristine compound file development

| **Study (reference)** | **Dose** | **Cohort** | **N** | **Age**  **(years)** | **BMI**  **(kg/m^2^)** |
| --- | --- | --- | --- | --- | --- |
| Adult (39) | 2 mg, 15 min iv infusion | Cancer patients  (0.50 female) | 6 | 40-54 | 17-32 |
| Adult mass balance study (35) | 2 mg, 1 min iv bolus | Cancer patients  (0.50 female) | 4 | 43-58 | 38-44 |
| Adult CYP3A5 EM and PM (36) | 2 mg, 15 min iv infusion | Cancer patients  (0.47 female) | 5 | 20-90 | NR |
| Pediatric (40) | 1.5 mg/m^2^ (2 mg max) iv bolus | Cancer patients  (0.37 female) | 30 | 1-17 | 0.21-2.1* |
| Pediatric (41) | 1-1.5 mg/m^2^ (2 mg max) iv bolus | Cancer patients  (0.50 female) | 8 | 0.4-12 | 0.79 (0.22) |

*BSA (m^2^) reported

iv: intravenous, min: minute, EM: extensive metabolizer, PM: poor metabolizer, NR: not reported.

Table S10	Input Parameters of the Vincristine Pediatric PBPK Model

| Parameter | Value | Assumption(s) and references |
| --- | --- | --- |
| Physicochemical properties and blood binding | | |
| Compound type | Small molecule |  |
| Molecular weight | 824.96 | (36) |
| Log P | 2.82 | (36) |
| Charge | Monoprotic acid | (36) |
| pKa 1 | 5.15 | (36) |
| B/P ratio | 1.2 | (36) |
| f_u_ | 0.51 | (36) |
| Plasma binding protein | AGP | (36) |
| **Absorption** | | |
| Absorption Model | ADAM |  |
| P_eff,man_ (10^-4^ cm/s) | 0.206 | Predicted by Simcyp |
| P_trans,0_ (10^-6^ cm/s) | 15.4 | Predicted by MechP_eff_ model with method 2 |
| Intrinsic solubility (mg/mL) | 0.198 | Predicted by Simcyp |
|  |  |  |
| Distribution | | |
| Distribution model | Full PBPK |  |
| Prediction method of V_ss_ | Method 3 | Apply sub-cellular distribution model “on” |
| V_ss_ (L/kg) | 16.0 | Simcyp predicted and literature V_dss_ 15.8 L/kg (38) |
| K_p_ scalar | 82 | Optimized to match observed V_ss_ (38) |
| Elimination | | |
| CYP3A4 |  |  |
| Vmax (pmol/min/pmol isoform) | 1.49 | (34) |
| Km (μmol/L) | 19.5 | (36) |
| CYP3A5 |  |  |
| Vmax (pmol/min/pmol isoform) | 11 | (34) |
| Km (µM) | 18.5 | (36) |
| Percentage available for re-absorption (%) | 19.8 | Optimized to capture mass balance data (35) |
| CL_R_ (L/h) | 2.1 | Optimized to capture mass balance data (35) |
| Transporter | | |
| P-gp |  |  |
| CL_int,T_ (µL/min/million cells) | 4.5 | Calculated and matched to mass balance data (35, 36) |
| RAF/REF | 1.5 | (36) |
| CL_PD_ - liver  (mL/min/million hepatocytes) | 0.37 | (36) |
| fu_IW_ - liver | 0.036 | (36) |
| fu_EW_ - liver | 1.0 | (36) |

LogP: octanol:buffer partition coefficient, B/P ratio: blood-to-plasma partition ratio, fu: fraction unbound, AGP: α1-Acid glycoprotein, ADAM: advanced dissolution, absorption and metabolism model, P_eff,man_: Human jejunum effective permeability, P_trans,0_: membrane passive intrinsic transcellular permeability, Mech P_eff_: mechanistic passive reginal permeability V_ss_: volume of distribution at stead-state, K_p_ scalar: value applied to all tissue:plasma partition coefficients, V_max_: maximum rate of metabolism, Km: Michaelis constant, f_u,mic_: fraction of unbound drug in the in vitro microsomal incubation, CL_R_: renal clearance, CL_int_: intrinsic clearance, K_i_: concentration of inhibitor that supports half maximal inhibition

Table S11	Predicted and observed PK parameters for vincristine

|  | **AUCinf (ng·h/mL)** | | **CL (L/h)** | |
| --- | --- | --- | --- | --- |
|  | **Predicted** | **Observed** | **Predicted** | **Observed** |
| **2 mg adults** (39) | 64.9  (38.5) | 65.1  (15.5) | 31.8  (38.8) | 34.1  (13.4) |
| **2 mg adults – PM** | 105  (37.0) | NR | 19.8  (42.4) | NR |
| **2 mg adults – EM** | 41.5  (30.4) | NR | 46.7  (27.7) | NR |
| **1.5 mg/m^2^ pediatric** (40) | 75.8  (41.7) | 72.9  (21.8) | 20.5  (45.2)† | 17.9  (17.0)† |
| **1.5 mg/m^2^ pediatric** (41) | 57.3  (27.9)* | 57.0  (62.1)* | 19.1  (49.4) | 25.2  (56.4) |
| **GMFE** | **1.02** | | **1.17** | |

*AUC_0-24h_

†Reported as BSA normalized CL (h/L/m^2^)

PM, poor metabolizer, EM: extensive metabolizer, NR: not reported, GMFE: geometric mean fold error, AUC_inf_: area under the curve from 0 to infinity, C_max_: maximum concentration, CL: total clearance (dose/AUC)

data presented as arithmetic mean (%coefficient of variance)

Figure S5	Predicted and observed plasma concentration curve of vincristine in adult cancer patients who are poor metabolizers (PM) through CYP3A5

Observed data extracted from Reddy, et al. (36)

Black circles: observed clinical data, solid black line: simulated arithmetic mean, dark grey line: simulated 95th percentile, light grey line: simulated 5th percentile

Figure S6	Predicted and observed plasma concentration curve of vincristine in pediatric cancer patients

Observed data extracted from Barnett, et al. (40)

Black circles: observed clinical data, solid black line: simulated arithmetic mean, dark grey line: simulated 95th percentile, light grey line: simulated 5th percentile

# Edoxaban

The PBPK model for edoxaban was constructed based on previously reported PBPK models (42, 43). The clinical data utilized in the model building and verification of the edoxaban PBPK model are listed in Table S12. The major metabolite M4 was not included in the present model since M4 is not cleared by P-gp or CYP3A4; therefore, its inclusion was determined to be out of scope for use to verify P-gp inhibition by posaconazole and isavuconazole. The model was first built using single dose iv data from Matsushima, et. al.(44)  and single oral solution mass balance data from Bathala, et. al.(45). The model performance was verified with additional 60 mg single dose oral data and observed DDI data with CYP3A4 and P-gp inhibitors ketoconazole, cyclosporine, verapamil, and quinidine. The final parameters used in the adult edoxaban PBPK model can be found in Table S13. A summary of observed and simulated PK parameters is listed in Table S14. A summary of model performance in DDI studies can be found in Table S15. Visual model performance for 60 mg of edoxaban can be found in Figure S7.

The model was built using ADAM absorption model with Mech P_eff_ model method 1 prediction, full PBPK for distribution, permeability limited liver model for P-gp transport with EHRC. V_ss_ was predicted using Method 2 (Rodgers et al.(46)) and a K_p_ scalar of 1.75 to capture literature reported V_ss_ (107 L) in healthy volunteers receiving 30 mg iv edoxaban (44). Model predicted V_ss_ for 30 mg iv edoxaban was 110 L.

CL_int_ through CYP3A4 was calculated using the built-in Simcyp retrograde calculator, assuming 10% metabolism through CYP3A4(43, 47), CL_iv_ of 21.8 L/h, and renal clearance (CL_R_) of 10.7 L/h (44). The value of CL_int,CYP3A4_ was verified with Simcyp mass balance analysis and reported clearance of metabolites M6 (2.11%) and M8 (0.34%) from the mass balance study(45). Secondarily, CYP3A4 contribution was verified with several clinical DDI studies. Clearance through esterase CES1 was estimated based on the recovery of metabolite M4 (3.5%) from the mass balance study(45). Kinetic clearance through P-gp was included based on published literature and the % available for reabsorption was estimated based on mass balance recovery of unchanged edoxaban in the feces(42, 43, 45). Additional systemic clearance was included to capture clearance of edoxaban through methods not included in the PBPK model (hydrolysis and other unidentified pathways). The PBPK model overestimated the percentage of unchanged edoxaban in the urine (1.42-fold higher) when utilizing the published value of 10.7 L/h. However, it was determined not to impact the predicted DDI with posaconazole and isavuconazole; therefore, it was left unmodified from the published value. A summary of observed and predicted mass balance data can be found in Table S16.

Based on the performance of the edoxaban PBPK model in capturing the observed clinical PK data, observed DDI studies, and mass balance data, it was determined that edoxaban was well verified to be utilized to predict P-gp inhibition by posaconazole and isavuconazole.

Table S12.	Clinical data utilized in the edoxaban compound file development

| **Study (reference)** | **Dose** | **Cohort** | **N** | **Age**  **(years)** | **BMI**  **(kg/m^2^)** |
| --- | --- | --- | --- | --- | --- |
| Single dose (44) | 30 mg iv over 30 min | Healthy  (0.08 female) | 35 | 18-45 | 21-32 |
| Single dose  (44, 47) | 60 mg oral | Healthy  (0.08 female) | 35 | 18-45 | 21-32 |
| Mass balance (45) | 60 mg oral | Healthy male | 6 | 30-55 | 19-29 |
| DDI ketoconazole (48) | day 1-7: 400 mg oral ketoconazole daily  day 4: 60 mg oral edoxaban | Healthy  (0.125 female) | 39 | 18-45 | 18-32 |
| DDI cyclosporine (48) | Single oral dose 500 mg cyclosporine + 60 mg edoxaban | Healthy  (0.125 female) | 39 | 18-45 | 18-32 |
| DDI verapamil (49) | day 1-11: 240 mg SR verapamil  day 10: 60 mg oral edoxaban | Healthy  (0.176 female) | 30 | 31.4 (8.3) | 79.0 (12.8)† |
| DDI quinidine (49) | day 1: 300 mg quinidine  day 2-3: 300 mg oral quinidine Q8H  day 4: 300 mg quinidine  day 3: 60 mg oral edoxaban | Healthy  (16.7 female) | 29 | 30.9 (7.5) | 79.7 (12.0)† |
| DDI quinidine (44) | day 1-3: 300 mg oral quinidine Q8H  day 1: single 30 mg iv edoxaban (over 30 min) | Healthy  (0.08 female) | 28 | 18-45 | 21-32 |

*BSA (m^2^) reported

†weight in kg, mean(SD)

iv: intravenous, min: minute, EM: extensive metabolizer, PM: poor metabolizer, NR: not reported.

Table S13	Input Parameters of the Edoxaban PBPK Model

| Parameter | Value | Assumption(s) and references |
| --- | --- | --- |
| Physicochemical properties and blood binding | | |
| Compound type | Small molecule |  |
| Molecular weight | 548.6 | (42) |
| Log P | 1.61 | (42) |
| Charge | Monoprotic base | (42) |
| pKa 1 | 6.7 | (42) |
| B/P ratio | 0.96 | (42) |
| f_u_ | 0.45 | (42) |
| Plasma binding protein | HSA | (42) |
| **Absorption** | | |
| Absorption Model | ADAM |  |
| f_uGut_ | 0.236 | Predicted by Simcyp |
| P_eff,man_ (10^-4^ cm/s) | 4.61 | Predicted by Simcyp using MechP_eff_ model method 1. P_ttrans,O_ = 112.4 10^-6^ cm/s |
| pH-Solubility (mg/mL) |  | (42, 43) |
| pH 3 | 4.4 |  |
| pH 4 | 4.2 |  |
| pH 5 | 1.8 |  |
| pH 6 | 0.54 |  |
| pH 7 | 0.14 |  |
| Formulation | Immediate Release | Diffusion layer model |
| Transit Time | Segregated transit time |  |
| Distribution | | |
| Distribution model | Full PBPK |  |
| Prediction method of V_ss_ | Method 2 |  |
| V_ss_ (L/kg) | 1.42 | Simcyp predicted |
| K_p_ scalar | 1.75 | Optimized to match observed V_ss_ (44) |
| Elimination | | |
| CYP3A4 CL_int_ (µL/min/pmol isoform) | 0.0034 | Calculated with retrograde calculator  (CL_iv_ 21.8 L/h, f_CL,Bile_ 20%, f_m,CYP3A4_ 10%)(47) |
| CES1 (µL/min/mg protein) | 0.29 | Optimized to match M4 recovery (3.5%) from mass balance study (45) |
| Percentage available for re-absorption (%) | 75 | (43) |
| CL_R_ (L/h) | 10.7 | (44) |
| Additional systemic clearance (L/h) | 1.4 | non-CYP3A4 and CES1 clearance (45) |
| Transporter | | |
| P-gp |  |  |
| J_max_ (pmol/min) | 108 | (42) |
| K_m_ (µM) | 9.1 |  |
| RAF/REF | 0.99 | Set to match default value for Caco-2 cells |
| CL_PD_ - liver (mL/min/million hepatocytes) | 1.01 | Calculated by Simcyp using Mech P_eff_ |

LogP: octanol:buffer partition, B/P ratio: blood-to-plasma partition, f_u_: fraction unbound, HSA: Human Serum Albumin, ADAM: advanced dissolution, absorption and metabolism, f_uGut_: fraction unbound in the gut, P_eff,man_: Human jejunum effective permeability, Mech P_eff_: mechanistic passive reginal permeability, V_ss_: volume of distribution at stead-state, K_p_ scalar: value applied to all tissue:plasma partition coefficients, CL_R_: renal clearance, CL_int_: intrinsic clearance, CL_iv_: intravenous clearance, f_m,CYP3A4_: fraction metabolized CYP3A4, J_max_: in vitro maximum rate of transporter-mediated efflux, K_m_: Michaelis constant, RAF/REF: Relative activity factor or relative expression factor, CL_PD_: passive diffusion clearance

Table S14	Predicted and observed PK parameters for edoxaban in adults

|  | **AUC_inf_ (ng·h/mL)** | | **C_max_ (ng/mL)** | | **CL/F (L/h)** | |
| --- | --- | --- | --- | --- | --- | --- |
|  | **Predicted** | **Observed** | **Predicted** | **Observed** | **Predicted** | **Observed** |
| **30 mg IV** | 1,200  (26.2) | 1,320  (14.4) | 518  (15.4) | 424  (27.1) | 26.9  (28.7) | 21.8  (13.9)* |
| **60 mg PO** | 1,780  (35.5) | 1,770  (24.6) | 304  (38.9) | 256  (34.3) | 39.5  (52.0) | 35.7  (21.4) |
| **60 mg PO (MB study)** | 2,090  (30.8) | 1,600  (19.4) | 367  (35.9) | 332  (32.5) | 32.0  (37.0) | 37.6  (NR) |
| **GMFE** | **1.14** | | **1.17** | | **1.17** | |

*CL

NR: not reported, GMFE: geometric mean fold error, AUC_inf_: area under the curve from 0 to infinity, C_max_: maximum concentration, CL: total clearance (dose/AUC)

data presented as arithmetic mean (%coefficient of variance)

Figure S7	Predicted and observed plasma concentration curve of oral edoxaban in adult healthy volunteers

Observed data extracted from Parasrampuria, et. al.(47)

Black circles: observed clinical data, solid black line: simulated arithmetic mean, dark grey line: simulated 95th percentile, light grey line: simulated 5th percentile

Table S15	Summary of predicted by the PBPK model and observed PK parameters (AUC and C_max_ ratio) of DDI for edoxaban as the victim

|  | **Without Inhibitor** | | **With Inhibitor** | |  | |
| --- | --- | --- | --- | --- | --- | --- |
|  | **AUC_inf_ (ng*h/mL)** | **C_max_ (ng/mL)** | **AUC_inf_ (ng*h/mL)** | **C_max_ (ng/mL)** | **AUC_inf_ ratio** | **C_max_ Ratio** |
| **400 mg ketoconazole**(48) | | | | | | |
| **Observed** | 1,650 | 245 | 3,000 | 441 | **1.87** | **1.89** |
| **90% CI** |  |  |  |  | (1.76, 1.98) | (1.68, 2.12) |
| **Predicted** | 1,640 | 256 | 3,480 | 504 | **2.12** | **1.97** |
| **90% CI** |  |  |  |  | (2.07, 2.18) | (1.97, 2.01) |
| **Criteria** |  |  |  |  | **1.17 - 2.99** | **1.18 - 3.03** |
| **500 mg cyclosporine** (48) | | | | | | |
| **Observed** | 1,830 | 246 | 3,130 | 411 | **1.73** | **1.74** |
| **90% CI** |  |  |  |  | (1.63, 1.83) | (1.56, 1.93) |
| **Predicted** | 1,640 | 257 | 2,350 | 358 | **1.44** | **1.39** |
| **90% CI** |  |  |  |  | (1.41, 1.46) | (1.36, 1.42) |
| **Criteria** |  |  |  |  | **1.10 - 2.71** | **1.11 - 2.73** |
| **240 mg verapamil SR**(49) ***** | | | | | | |
| **Observed** | 1,450 | 223 | 2,490 | 390 | **1.53** | **1.53** |
| **90% CI** |  |  |  |  | (1.41, 1.65) | 1.34, 1.76) |
| **Predicted** | 1,380 | 259 | 1,940 | 330 | **1.41** | **1.27** |
| **90% CI** |  |  |  |  | (1.38, 1.43) | (1.26, 1.29) |
| **Criteria** |  |  |  |  | **1.01 - 2.3** | **1.01 - 2.31** |
| **300 mg quinidine (oral edoxaban)**(49) ***** | | | | | | |
| **Observed** | 1,440 | 226 | 2,110 | 320 | **1.77** | **1.85** |
| **90% CI** |  |  |  |  | (1.65, 1.89) | (1.65, 2.09) |
| **Predicted** | 1,590 | 289 | 2,520 | 494 | **1.59** | **1.71** |
| **90% CI** |  |  |  |  | (1.56, 1.61) | (1.67, 1.74) |
| **Criteria** |  |  |  |  | **1.12 - 2.79** | **1.16 - 2.95** |
| **300 mg quinidine (iv edoxaban)**(44) | | | | | | |
| **Observed** | 1,390 | 438 | 1,870 | 471 | **1.35** | **1.07** |
| **90% CI** |  |  |  |  | (1.28, 1.43) | (0.963, 1.20) |
| **Predicted** | 1,120 | 509 | 1,460 | 580 | **1.30** | **1.14** |
| **90% CI** |  |  |  |  | (1.29, 1.32) | (1.13, 1.14) |
| **Criteria** |  |  |  |  | **0.935 - 1.95** | **0.824 - 1.39** |

*AUC_0-24h_: area under the curve from 0 to 24 hour

AUC_inf_: area under the curve from 0 to infinity, C_max_: maximum concentration, CI: confidence interval, iv: intravenous

AUC ratio: AUC(with inhibitor)/AUC(without inhibitor), C_max_ ratio: C_max_(with inhibitor)/C_max_(without inhibitor)

Criteria determined by GUEST method assuming 20% variability in pharmacokinetic parameters(28)

Table S16	Edoxaban mass balance analysis from clinical study and predicted by the PBPK model

|  | **% Feces** | | **%Urine** | | **%Total Recovery** | |
| --- | --- | --- | --- | --- | --- | --- |
| **Analyte** | **Predicted** | **Observed** | **Predicted** | **Observed** | **Predicted** | **Observed** |
| % of dose | - | 62.2 | - | 35.4 | 100 | 97.6 |
| Edoxaban | 53.1 | 49.1 | 33.9 | 23.8 | 87.0 | 72.8 |
| CYP3A4 metabolism | - | 2.00 | - | 0.45 | 2.80 | 2.45 |
| CES1 metabolism | - | 1.93 | - | 1.56 | 5.32 | 3.49 |
| Additional CL | - | 0.05 | - | 3.22 | 4.58 | 3.27 |

# Isavuconazole

Table S17	Summary of PK studies used in the development and verification of the isavuconazole PBPK model

| **Study (reference)** | **Dose** | **Form** | **Cohort** | **N** | **Age**  **(years)** | **Weight**  **(kg)** |
| --- | --- | --- | --- | --- | --- | --- |
| adult SAD(13) | 100, 200, 400 mg | oral | healthy males | 15 | 22-45 | 57-88 |
| adult SAD(13) | 40, 80, 160 mg | iv | healthy males | 18 | 23-45 | 57-89 |
| adult MAD(14) | day 1: 100 / 200 mg  day 2-14: 50 / 100 mg | oral | healthy males | 12 | 21-45 | 66-94 |
| adult MAD(14) | day 1: 80 / 160 mg  day 2-14: 40 / 80 mg | iv | healthy males | 12 | 26-45 | 65-101 |
| pediatric(15) | 10 mg/kg (200 mg max)  day 1: Q8H  day3-28: daily | iv | children requiring antifungal prophylaxis | 26 | 1 to <18 | 9-104 |
| pediatric(15) | 10 mg/kg (200 mg max)  day 1: Q8H  day3-28: daily | oral | children requiring antifungal prophylaxis | 19 | 6 to <18 | 18-93 |

SAD: single ascending dose, MAD: multiple ascending dose, Q8H: every 8 hours

Information also provided in J-PKPD Isavuconazole PBPK manuscript (under peer review)

Table S18	Summary of DDI studies used in the development and verification of the isavuconazole PBPK model

| **Perpetrator** | **Perpetrator Dosing** | **Victim** | **Victim Dosing** | **Cohort** | **N** | **Age (yr)** | **Ref** |
| --- | --- | --- | --- | --- | --- | --- | --- |
| ketoconazole | day 1: 200 mg | isavuconazole | day 1: 400 mg | healthy males | 12 | 21-45 | (9) |
| ketoconazole | day 1-24: 200 mg Q12H | isavuconazole | day 4: 200 mg | Healthy  (0.33 female) | 12 | 19-53 | (9) |
| ketoconazole | day 1-36: 200 mg QD | isavuconazole | day 9: 400 mg day 10-22: 100 mg (2h lag) | healthy males | 26 | 19-64 | (9) |
| rifampicin | day 1-36: 600 mg QD | isavuconazole | day 9: 400 mg day 10-22: 100 mg (2h lag) | healthy males | 26 | 19-64 | (9) |
| isavuconazole | day 1: 400 mg day 2-28: 100 mg | warfarin | day 21: 10 mg (2h lag) | healthy males | 12 | 22-45 | (16) |
| isavuconazole | day 1-2: 200mg Q8H day 3-13: 200 mg QD | warfarin | day 5: 20 mg | healthy males | 20 | 23-54 | (16) |
| isavuconazole | day 1: 400 mg day 2-20: 100 mg QD | cyclosporine | day 15: 300 mg | healthy males | 26 | 22-43 | (17) |
| isavuconazole | day 1-2: 200mg Q8H day 3-8: 200 mg QD | cyclosporine | day 5: 300 mg | Healthy  (0.21 female) | 24 | 18-54 | (17) |
| isavuconazole | day 1-2: 200 mg Q8H day 3-11: 200 mg QD | midazolam | day 10: 3 mg | Healthy  (0.3 female) | 23 | 19-54 | (9) |
| isavuconazole | day 1-2: 200 mg Q8H day 3-12: 200 mg QD | digoxin | day 5: 0.5 mg | Healthy  (0.25 female) | 24 | 20-55 | (18) |
| isavuconazole | day 1-2: 200 mg Q8H day 3-7: 200 mg QD | dextromethorphan | day 5: 30 mg | Healthy  (0.29 female) | 24 | 21-53 | (19) |
| isavuconazole | day 1-2: 200 mg Q8H day 3-13: 200 mg daily | repaglinide | day 10: 0.5 mg | healthy males | 24 | 24-52 | (19) |
| isavuconazole | day 1-2: 200 mg Q8H day 3-8: 200 mg daily | ethinyl estradiol | day 5: 0.035 mg | healthy females | 24 | 51-65 | (9) |
| isavuconazole | day 1-2: 200 mg Q8H day 3-13: 200 mg daily | bupropion | day 8: 100 mg | Healthy  (0.46 female) | 24 | 21-55 | (19) |
| Isavuconazole | day 1: 200 mg Q8H | rivaroxaban | day 1: 25 µg 1 hr after | Healthy  (0.42 femal) | 12 | 20-48 | (20) |
| isavuconazole | day 1: 200 mg Q8H | edoxaban | day 1: 50 µg 1 hr after | Healthy  (0.42 female) | 12 | 20-48 | (20) |

yr: year, QXH: every X hours, QD: every day, Information also provided in J-PKPD Isavuconazole PBPK manuscript (under peer review)

# Sensitivity Analysis

Table S19.	Sensitivity analysis for isavuconazole and posaconazole K_i,P-gp,liver_ and K_i,CYP3A4_

|  | **ISA** | **POSA** | **ISA** | | | | **POSA** | |
| --- | --- | --- | --- | --- | --- | --- | --- | --- |
| **K_i,P-gp_**  **(µmol/L)** | **AUC ratio** | **AUC ratio** | **K_i,CYP3A5_**  **(µmol/L)*** | **AUC ratio** | **K_i,CYP3A4_**  **(µmol/L)** | **AUC ratio** | **K_i,CYP3A4_**  **(µmol/L)** | **AUC ratio** |
| 0.0010 | 2.26 | 5.40 | 0.019 | 2.10 | 0.019 | 3.25 | 0.019 | 6.68 |
| 0.0017 | 2.23 | 5.38 | 0.032 | 1.82 | 0.032 | 2.87 | 0.031 | 6.51 |
| 0.0028 | 2.18 | 5.34 | 0.053 | 1.59 | 0.053 | 2.53 | 0.051 | 6.25 |
| 0.0046 | 2.11 | 5.29 | 0.088 | 1.41 | 0.088 | 2.25 | 0.086 | 5.88 |
| 0.0077 | 2.02 | 5.20 | 0.147 | 1.29 | 0.15 | 2.04 | 0.14 | 5.41 |
| 0.013 | 1.89 | 5.06 | 0.245 | 1.21 | 0.25 | 1.89 | 0.24 | 4.84 |
| 0.022 | 1.74 | 4.85 | 0.409 | 1.16 | 0.41 | 1.79 | 0.40 | 4.24 |
| 0.036 | 1.59 | 4.56 | 0.683 | 1.12 | 0.68 | 1.73 | 0.66 | 3.66 |
| 0.060 | 1.45 | 4.18 | 1.14 | 1.10 | 1.14 | 1.69 | 1.11 | 3.16 |
| 0.100 | 1.33 | 3.72 | 1.90 | 1.09 | 1.90 | 1.67 | 1.85 | 2.77 |

ISA: isavuconazole, POSA: posaconazole

Values above and below the thick black line are respectively 10-fold lower and higher than the final model value

*Sensitivity analysis for K_i,CYP3A5_ was carried out in a CYP3A5 extensive metabolizer

Table S20	Sensitivity analysis for vincristine V_max,CYP3A_ and CL_int,P-gp,intestine_ in DDI with posaconazole

| **V_max,CYP3A5_**  **(pmol/min/pmol isoform)*** | **AUC ratio** | **V_max,CYP3A4_**  **(pmol/min/pmol isoform)** | **AUC ratio** | **CL_int,P-gp,liver_**  **(µL/min/million cells)** | **AUC ratio** |
| --- | --- | --- | --- | --- | --- |
| 1.10 | 2.95 | 0.15 | 4.99 | 0.45 | 3.01 |
| 1.83 | 2.37 | 0.25 | 5.01 | 0.75 | 3.20 |
| 3.06 | 1.88 | 0.41 | 5.05 | 1.25 | 3.51 |
| 5.11 | 1.53 | 0.69 | 5.09 | 2.09 | 3.98 |
| 8.52 | 1.29 | 1.15 | 5.13 | 3.48 | 4.69 |
| 14.2 | 1.15 | 1.92 | 5.13 | 5.81 | 5.65 |
| 23.7 | 1.07 | 3.21 | 5.02 | 9.69 | 6.81 |
| 39.5 | 1.03 | 5.35 | 4.75 | 16.2 | 7.97 |
| 65.9 | 1.01 | 8.93 | 4.29 | 27.0 | 8.77 |
| 110 | 1.01 | 14.9 | 3.69 | 45.0 | 8.89 |

Values above and below the thick black line are respectively 10-fold lower and higher than the final model value

*Sensitivity analysis for V_max,CYP3A5_ was carried out in a CYP3A5 extensive metabolizer

# References

1. Malik P, Mian P. 2025. Physiologically Based Pharmacokinetic Modeling to Refine Dosing of Posaconazole in Young Children. Clin Ther 47:261–270.

2. Farhan N, Dahal UP, Wahlstrom J. 2024. Development and Evaluation of Ontogeny Functions of the Major UDP‐Glucuronosyltransferase Enzymes to Underwrite Physiologically Based Pharmacokinetic Modeling in Pediatric Populations. J Clin Pharmacol 64:1222–1235.

3. Salem F, Johnson TN, Abduljalil K, Tucker GT, Rostami-Hodjegan A. 2014. A Re-evaluation and Validation of Ontogeny Functions for Cytochrome P450 1A2 and 3A4 Based on In Vivo Data. Clin Pharmacokinet 53:625–636.

4. Salem F, Johnson TN, Abduljalil K, Tucker GT, Rostami-Hodjegan A. 2015. Erratum to: A Re-evaluation and Validation of Ontogeny Functions for Cytochrome P450 1A2 and 3A4 Based on In Vivo Data. Clin Pharmacokinet 54:671–671.

5. Upreti VV, Wahlstrom JL. 2016. Meta‐analysis of hepatic cytochrome P450 ontogeny to underwrite the prediction of pediatric pharmacokinetics using physiologically based pharmacokinetic modeling. J Clin Pharmacol 56:266–283.

6. Codaccioni M, Southall RL, Dinh J, Johnson TN. 2024. Prediction of Pediatric Pharmacokinetics for CYP3A4 Metabolized Drugs: Comparison of the Performance of Two Hepatic Ontogeny Within a Physiologically Based Pharmacokinetic Model. J Clin Pharmacol 64:1083–1094.

7. Hens B, Pathak SM, Mitra A, Patel N, Liu B, Patel S, Jamei M, Brouwers J, Augustijns P, Turner DB. 2017. In Silico Modeling Approach for the Evaluation of Gastrointestinal Dissolution, Supersaturation, and Precipitation of Posaconazole. Mol Pharm 14:4321–4333.

8. Chen K-F, Chan L-N, Lin YS. 2020. PBPK modeling of CYP3A and P-gp substrates to predict drug–drug interactions in patients undergoing Roux-en-Y gastric bypass surgery. J Pharmacokinet Pharmacodyn 47:493–512.

9. Kersemaekers WM, Iersel T van, Nassander U, O’Mara E, Waskin H, Caceres M, Iersel MLPS van. 2014. Pharmacokinetics and Safety Study of Posaconazole Intravenous Solution Administered Peripherally to Healthy Subjects. Antimicrob Agents Chemother 59:1246–1251.

10. Krishna G, Ma L, Martinho M, O’Mara E. 2012. Single-Dose Phase I Study To Evaluate the Pharmacokinetics of Posaconazole in New Tablet and Capsule Formulations Relative to Oral Suspension. Antimicrob Agents Chemother 56:4196–4201.

11. Krieter P, Flannery B, Musick T, Gohdes M, Martinho M, Courtney R. 2004. Disposition of Posaconazole following Single-Dose Oral Administration in Healthy Subjects. Antimicrob Agents Chemother 48:3543–3551.

12. Krishna G, Moton A, Ma L, Medlock MM, McLeod J. 2008. Pharmacokinetics and Absorption of Posaconazole Oral Suspension under Various Gastric Conditions in Healthy Volunteers. Antimicrob Agents Chemother 53:958–966.

13. Pandav T, Devare T, Pant P, Mali A. 2023. Development of posaconazole delayed release formulation for mucormycosis by hot melt extrusion using QBD approach. J Adv Sci Res 14:28–39.

14. Krishna G, Ma L, Martinho M, Preston RA, O’Mara E. 2012. A new solid oral tablet formulation of posaconazole: a randomized clinical trial to investigate rising single- and multiple-dose pharmacokinetics and safety in healthy volunteers. J Antimicrob Chemother 67:2725–2730.

15. Silva MI, Khadra I, Pyper K, Halbert GW. 2023. Structured solubility behaviour in fed simulated intestinal fluids. Eur J Pharm Biopharm 193:58–73.

16. Lempers VJC, Heuvel JJMW van den, Russel FGM, Aarnoutse RE, Burger DM, Brüggemann RJ, Koenderink JB. 2016. Inhibitory Potential of Antifungal Drugs on ATP-Binding Cassette Transporters P-Glycoprotein, MRP1 to MRP5, BCRP, and BSEP. Antimicrob Agents Chemother 60:3372–3379.

17. Maertens J, Cornely OA, Ullmann AJ, Heinz WJ, Krishna G, Patino H, Caceres M, Kartsonis N, Waskin H, Robertson MN. 2014. Phase 1B Study of the Pharmacokinetics and Safety of Posaconazole Intravenous Solution in Patients at Risk for Invasive Fungal Disease. Antimicrob Agents Chemother 58:3610–3617.

18. Groll AH, Abdel-Azim H, Lehrnbecher T, Steinbach WJ, Paschke A, Mangin E, Winchell GA, Waskin H, Bruno CJ. 2020. Pharmacokinetics and safety of posaconazole intravenous solution and powder for oral suspension in children with neutropenia: an open-label, sequential dose-escalation trial. Int J Antimicrob Agents 56:106084.

19. Krishna G, Ma L, Prasad P, Moton A, Martinho M, O’Mara E. 2012. Effect of posaconazole on the pharmacokinetics of simvastatin and midazolam in healthy volunteers. Expert Opin Drug Metab Toxicol 8:1–10.

20. Krishna G, Moton A, Ma L, Savant I, Martinho M, Seiberling M, McLeod J. 2009. Effects of oral posaconazole on the pharmacokinetic properties of oral and intravenous midazolam: A phase I, randomized, open-label, crossover study in healthy volunteers. Clin Ther 31:286–298.

21. Rohr BS, Foerster KI, Blank A, Burhenne J, Mahmoudi M, Haefeli WE, Mikus G. 2022. Perpetrator Characteristics of Azole Antifungal Drugs on Three Oral Factor Xa Inhibitors Administered as a Microdosed Cocktail. Clin Pharmacokinet 61:97–109.

22. Vemuri VD, Lankalapalli S, Guntaka PCR. 2022. Posaconazole-amino acid cocrystals for improving solubility and oral bioavailability while maintaining antifungal activity and low In vivo toxicity. J Drug Deliv Sci Technol 74:103491.

23. Lignell A, Löwdin E, Cars O, Chryssanthou E, Sjölin J. 2011. Posaconazole in Human Serum: a Greater Pharmacodynamic Effect than Predicted by the Non-Protein-Bound Serum Concentration. Antimicrob Agents Chemother 55:3099–3104.

24. Kersemaekers WM, Iersel T van, Nassander U, O’Mara E, Waskin H, Caceres M, Iersel MLPS van. 2016. Erratum for Kersemaekers et al., Pharmacokinetics and Safety Study of Posaconazole Intravenous Solution Administered Peripherally to Healthy Subjects. Antimicrob Agents Chemother 60:4426–4426.

25. Sime FB, Stuart J, Butler J, Starr T, Wallis SC, Pandey S, Lipman J, Roberts JA. 2018. Pharmacokinetics of Intravenous Posaconazole in Critically Ill Patients. Antimicrob Agents Chemother 62:10.1128/aac.00242-18.

26. Blennow O, Eliasson E, Pettersson T, Pohanka A, Szakos A, El-Serafi I, Hassan M, Ringdén O, Mattsson J. 2014. Posaconazole Concentrations in Human Tissues after Allogeneic Stem Cell Transplantation. Antimicrob Agents Chemother 58:4941–4943.

27. Lempers VJ, Martial LC, Schreuder MF, Blijlevens NM, Burger DM, Aarnoutse RE, Brüggemann RJ. 2015. Drug-interactions of azole antifungals with selected immunosuppressants in transplant patients: strategies for optimal management in clinical practice. Curr Opin Pharmacol 24:38–44.

28. Guest EJ, Aarons L, Houston JB, Rostami-Hodjegan A, Galetin A. 2011. Critique of the Two-Fold Measure of Prediction Success for Ratios: Application for the Assessment of Drug-Drug Interactions. Drug Metab Dispos 39:170–173.

29. Groll AH, Desai A, Han D, Howieson C, Kato K, Akhtar S, Kowalski D, Lademacher C, Lewis W, Pearlman H, Mandarino D, Yamazaki T, Townsend R. 2017. Pharmacokinetic Assessment of Drug‐Drug Interactions of Isavuconazole With the Immunosuppressants Cyclosporine, Mycophenolic Acid, Prednisolone, Sirolimus, and Tacrolimus in Healthy Adults. Clin Pharmacol Drug Dev 6:76–85.

30. Michael C, Bierbach U, Frenzel K, Lange T, Basara N, Niederwieser D, Mauz-Körholz C, Preiss R. 2010. Voriconazole Pharmacokinetics and Safety in Immunocompromised Children Compared to Adult Patients. Antimicrob Agents Chemother 54:3225–3232.

31. Mori M, Fukushima K, Miharu M, Miharu M, Goto H, Yoshida M, Yoshida M, Shoji S. 2013. A retrospective analysis of voriconazole pharmacokinetics in Japanese pediatric and adolescent patients. J Infect Chemother 19:174–179.

32. 2014. VinCRIStine Sulfate FDA label.

33. Dennison JB, Jones DR, Renbarger JL, Hall SD. 2007. Effect of CYP3A5 Expression on Vincristine Metabolism with Human Liver Microsomes. J Pharmacol Exp Ther 321:553–563.

34. Dennison JB, Kulanthaivel P, Barbuch RJ, Renbarger JL, Ehlhardt WJ, Hall SD. 2006. Selective metabolism of vincristine in vitro by CYP3A5. Drug Metab Dispos 34:1317–1327.

35. Bender RA, Castle MC, Margileth DA, Oliverio VT. 1977. The pharmacokinetics of [3H]‐vincristine in man. Clin Pharmacol Ther 22:430–438.

36. Reddy VP, Fretland AJ, Zhou D, Sharma S, Chen B, Vishwanathan K, McGinnity DF, Xu Y, Ware JA. 2021. Mechanistic physiology-based pharmacokinetic modeling to elucidate vincristine-induced peripheral neuropathy following treatment with novel kinase inhibitors. Cancer Chemother Pharmacol 88:451–464.

37. Lee CM, Zane NR, Veal G, Thakker DR. 2019. Physiologically Based Pharmacokinetic Models for Adults and Children Reveal a Role of Intracellular Tubulin Binding in Vincristine Disposition. CPT: Pharmacomet Syst Pharmacol 8:759–768.

38. Crom WR, Graaf SSN de, Synold T, Uges DRA, Bloemhof H, Rivera G, Christensen ML, Mahmoud H, Evans WE. 1994. Pharmacokinetics of vincristine in children and adolescents with acute lymphocytic leukemia. J Pediatr 125:642–649.

39. Villikka K, Kivistö KT, Mäenpää H, Joensuu H, Neuvonen PJ. 1999. Cytochrome P450‐inducing antiepileptics increase the clearance of vincristine in patients with brain tumors. Clin Pharmacol Ther 66:589–593.

40. Barnett S, Hellmann F, Parke E, Makin G, Tweddle DA, Osborne C, Hempel G, Veal GJ. 2022. Vincristine dosing, drug exposure and therapeutic drug monitoring in neonate and infant cancer patients. Eur J Cancer 164:127–136.

41. Israels T, Damen CWN, Cole M, Geloven N van, Boddy AV, Caron HN, Beijnen JH, Molyneux EM, Veal GJ. 2010. Malnourished Malawian patients presenting with large Wilms tumours have a decreased vincristine clearance rate. Eur J Cancer 46:1841–1847.

42. Xu R, Liu W, Ge W, He H, Jiang Q. 2023. Physiologically‐based pharmacokinetic pharmacodynamic parent‐metabolite model of edoxaban to predict drug–drug‐disease interactions: M4 contribution. CPT: Pharmacomet Syst Pharmacol 12:1093–1106.

43. Kato T, Mikkaichi T, Yoshigae Y, Okudaira N, Shimizu T, Izumi T, Ando S, Matsumoto Y. 2021. Quantitative analysis of an impact of P-glycoprotein on edoxaban’s disposition using a human physiologically based pharmacokinetic (PBPK) model. Int J Pharm 597:120349.

44. Matsushima N, Lee F, Sato T, Weiss D, Mendell J. 2013. Bioavailability and Safety of the Factor Xa Inhibitor Edoxaban and the Effects of Quinidine in Healthy Subjects. Clin Pharmacol Drug Dev 2:358–366.

45. Bathala MS, Masumoto H, Oguma T, He L, Lowrie C, Mendell J. 2012. Pharmacokinetics, Biotransformation, and Mass Balance of Edoxaban, a Selective, Direct Factor Xa Inhibitor, in Humans. Drug Metab Dispos 40:2250–2255.

46. Rodgers T, Rowland M. 2007. Mechanistic Approaches to Volume of Distribution Predictions: Understanding the Processes. Pharm Res 24:918–933.

47. Parasrampuria DA, Truitt KE. 2016. Pharmacokinetics and Pharmacodynamics of Edoxaban, a Non-Vitamin K Antagonist Oral Anticoagulant that Inhibits Clotting Factor Xa. Clin Pharmacokinet 55:641–655.

48. Parasrampuria DA, Mendell J, Shi M, Matsushima N, Zahir H, Truitt K. 2016. Edoxaban drug–drug interactions with ketoconazole, erythromycin, and cyclosporine. Br J Clin Pharmacol 82:1591–1600.

49. Mendell J, Zahir H, Matsushima N, Noveck R, Lee F, Chen S, Zhang G, Shi M. 2013. Drug-Drug Interaction Studies of Cardiovascular Drugs Involving P-Glycoprotein, an Efflux Transporter, on the Pharmacokinetics of Edoxaban, an Oral Factor Xa Inhibitor. Am J Cardiovasc Drugs 13:331–342.
